# Correction: Gandhi et al. Ultrasound-Mediated Blood–Brain Barrier Disruption for Drug Delivery: A Systematic Review of Protocols, Efficacy, and Safety Outcomes from Preclinical and Clinical Studies. *Pharmaceutics* 2022, *14*, 833

**DOI:** 10.3390/pharmaceutics15010005

**Published:** 2022-12-20

**Authors:** Kushan Gandhi, Anita Barzegar-Fallah, Ashik Banstola, Shakila B. Rizwan, John N. J. Reynolds

**Affiliations:** 1 Department of Anatomy, School of Biomedical Sciences, University of Otago, Dunedin 9016, New Zealand; 2 Brain Health Research Centre, University of Otago, Dunedin 9016, New Zealand; 3 School of Pharmacy, University of Otago, Dunedin 9016, New Zealand

**Error in Table**

In the original publication [1], there was a mistake in Table 3 as published. The mistakes pertained to the manufacturer information listed in the rows corresponding to the Optison^®^ and SonoVue®/Lumason^®^ microbubbles. We acknowledge that some manufacturer information was outdated, and no longer currently accurate, e.g., the mean bubble diameters and concentrations. In addition, some information was accidentally misclassified between the two microbubbles, e.g., the gas core and shell composition. The corrected Table 3 appears below. The authors state that the scientific conclusions are unaffected. This correction was approved by the Academic Editor. The original publication has also been updated.

Original:

**Table 3:** Comparison of five major commercially available microbubble formulations used in studies for ultrasound-mediated BBB disruption (information sourced from manufacturer) and typical doses.

Agent	Manufacturer	Shell Composition	Gas Core Composition	Mean Bubble Diameter (µm)	Bubble Concentration (Bubbles/mL)	Use in Identified Studies
Definity^®^/Luminity^®^	Lantheus Medical Imaging	Lipid	C_3_F_8_	1.1–3.3	1.2 × 10^10^	Used in *n* = 42 preclinical studies (typical doses: 10–20 µL/kg) and *n* = 6 clinical studies (typical dose: 4 µL/kg)
Optison^®^	GE Healthcare	Lipid	SF_6_	2.0–4.5	5–8 × 10^8^	Used in *n* = 14 preclinical studies (typical doses: 50–100 µL/kg but significantly varied in mouse studies)
SonoVue^®^/Lumason^®^	Bracco Diagnostics	Lipid-protein	C_3_F_8_	2.5	1–5 × 10^8^	Used in *n* = 29 preclinical studies (typical doses 25–150 µL/kg) and *n* = 2 clinical studies (typical dose: 100 µL/kg)
Usphere Prime^®^	Trust Bio-sonics	Lipid	C_3_F_8_	1.0	2.8 × 10^10^	Used in *n* = 1 preclinical study
Sonazoid^®^	GE Healthcare	Lipid	C_4_F_10_	2.0–3.0	9 × 10^8^	Used in *n* = 1 preclinical study

Corrected:

**Table 3:** Comparison of five major commercially available microbubble formulations used in studies for ultrasound-mediated BBB disruption (information sourced from manufacturer) and typical doses.

Agent	Manufacturer	Shell Composition	Gas Core Composition	Mean Bubble Diameter (µm)	Bubble Concentration (Bubbles/mL)	Use in Identified Studies
Definity^®^/Luminity^®^	Lantheus Medical Imaging	Lipid	C_3_F_8_	1.1–3.3	1.2 × 10^10^	Used in *n* = 42 preclinical studies (typical doses: 10–20 µL/kg) and *n* = 6 clinical studies (typical dose: 4 µL/kg)
Optison^®^	GE Healthcare	Protein	C_3_F_8_	3.0–4.5	5–8 × 10^8^	Used in *n* = 14 preclinical studies (typical doses: 50–100 µL/kg but significantly varied in mice studies)
SonoVue®/Lumason^®^	Bracco Diagnostics	Lipid	SF_6_	1.5–2.5	1.5–5.6 × 10^8^	Used in *n* = 29 preclinical studies (typical doses 25–150 µL/kg) and *n* = 2 clinical studies (typical dose: 100 µL/kg)
Usphere Prime^®^	Trust Bio-sonics	Lipid	C_3_F_8_	1.0	2.8 × 10^10^	Used in *n* = 1 preclinical study
Sonazoid^®^	GE Healthcare	Lipid	C_4_F_10_	2.0–3.0	9 × 10^8^	Used in *n* = 1 preclinical study
