# Supplementary material for: Ultrasound-Mediated Blood–Brain Barrier Disruption for Drug Delivery: A Systematic Review of Protocols, Efficacy, and Safety Outcomes from Preclinical and Clinical Studies
Source: Pharmaceutics. 2022 Apr 11;14(4):833. doi: 10.3390/pharmaceutics14040833 (PMC9029131; doi:10.3390/pharmaceutics14040833)
Supplement: Supplementary file 1 [file pharmaceutics-14-00833-s001.zip › pharmaceutics-1637448-supplementary/Revised Supplementary Materials/Table S1.pdf]

Table S1: PRISMA 2020 Checklist for reporting systematic reviews

Section and Topic	Item #	Checklist item	Location where item is reported
**TITLE**			
Title	1	Identify the report as a systematic review.	This study is a systematic review, without meta-analysis, of studies, conducted in both preclinical animal models and clinical human studies, as included in the title.
**ABSTRACT**			
Abstract	2	Provide a structured summary including, as applicable: background; objectives; data sources; study eligibility criteria, participants, and interventions; study appraisal and synthesis methods; results; limitations; conclusions and implications of key findings; systematic review registration number.	The applicable information is listed within the abstract section.<br><br>Background is summarised in the 'Background' section.<br>Objectives, study eligibility criteria and interventions are summarised in the 'Aim' section.<br>Data sources, study appraisal and synthesis methods are summarised in the 'Methods' section.<br>Participants and results are summarised in the 'Results' section.<br>Limitations, conclusions and implications are summarised in the 'Conclusions' section.<br>Given the nature of the available data, a systematic review registration number is not available, and the authors decided not to register it.
**INTRODUCTION**			
Rationale	3	Describe the rationale for the review in the context of existing knowledge.	The rationale is described in the 'Aim' section of the abstract as well as in the last paragraph of the 'Introduction' section.
Objectives	4	Provide an explicit statement of the objective(s) or question(s) the review addresses.	The rationale is described in the 'Aim' section of the abstract as well as in the last paragraph of the 'Introduction' section.<br><br>A known comparison, beyond a control not receiving US-BBBD is not possible. Intervention parameters and protocols related to the intervention and the outcomes of efficacy and safety have also been defined within the remaining manuscript.
**METHODS**			
Eligibility criteria	5	Specify the inclusion and exclusion criteria for the review and how studies were grouped for the syntheses.	The explicit inclusion and exclusion criteria, as well as how studies were grouped within the synthesis is explained in the 'Eligibility Criteria paragraph, in the materials and methods' section of the manuscript.
Information sources	6	Specify all databases, registers, websites, organisations, reference lists and other sources searched or consulted to identify studies. Specify the date when each source was last searched	Information sources, as well as dates of search were included in the 'Information Sources' paragraph of the

Section and Topic	Item #	Checklist item	Location where item is reported
		or consulted.	methods section.
Search strategy	7	Present the full search strategies for all databases, registers and websites, including any filters and limits used.	Search strategies were explained within the 'Information Sources' and 'Search' paragraphs of the methods section.
Selection process	8	Specify the methods used to decide whether a study met the inclusion criteria of the review, including how many reviewers screened each record and each report retrieved, whether they worked independently, and if applicable, details of automation tools used in the process.	Methods used to select each included study as well as reviewer(s) involved were summarised in the 'Study Selection' paragraph of the methods section, as well as in 'Figure 2" in the results section.
Data collection process	9	Specify the methods used to collect data from reports, including how many reviewers collected data from each report, whether they worked independently, any processes for obtaining or confirming data from study investigators, and if applicable, details of automation tools used in the process.	The data extraction and collection process, as well as the reviewer involved, is listed in the 'Data Items and Collection Process' paragraph of the methods section. No automation tolls were utilised in this process.
Data items	10a	List and define all outcomes for which data were sought. Specify whether all results that were compatible with each outcome domain in each study were sought (e.g. for all measures, time points, analyses), and if not, the methods used to decide which results to collect.	The data extraction and collection process, as well as the reviewer involved, is listed in the 'Data Items and Collection Process' paragraph of the methods section. No automation tolls were utilised in this process.
	10b	List and define all other variables for which data were sought (e.g. participant and intervention characteristics, funding sources). Describe any assumptions made about any missing or unclear information.	The data extraction and collection process, as well as the reviewer involved, is listed in the 'Data Items and Collection Process' paragraph of the methods section. No automation tolls were utilised in this process.
Study risk of bias assessment	11	Specify the methods used to assess risk of bias in the included studies, including details of the tool(s) used, how many reviewers assessed each study and whether they worked independently, and if applicable, details of automation tools used in the process.	Given the predominantly pre-clinical and early clinical stages of testing US-BBBD for drug delivery, a bias assessment was not possible.
Effect measures	12	Specify for each outcome the effect measure(s) (e.g. risk ratio, mean difference) used in the synthesis or presentation of results.	N/A – no quantitative analysis (e.g. meta-analysis) was conducted.
Synthesis methods	13a	Describe the processes used to decide which studies were eligible for each synthesis (e.g. tabulating the study intervention characteristics and comparing against the planned groups for each synthesis (item #5)).	N/A – no quantitative analysis (e.g. meta-analysis) was conducted.
	13b	Describe any methods required to prepare the data for presentation or synthesis, such as handling of missing summary statistics, or data conversions.	N/A – no quantitative analysis (e.g. meta-analysis) was conducted.
	13c	Describe any methods used to tabulate or visually display results of individual studies and syntheses.	Methods of tabulating and visually displaying results of individual studies is listed in the 'Data Items and Collection Process' paragraph of the methods section.
	13d	Describe any methods used to synthesize results and provide a rationale for the choice(s). If meta-analysis was performed, describe the model(s), method(s) to identify the presence and extent of statistical heterogeneity, and software package(s) used.	N/A – no quantitative analysis (e.g. meta-analysis) was conducted.
	13e	Describe any methods used to explore possible causes of heterogeneity among study results (e.g. subgroup analysis, meta-regression).	N/A – no quantitative analysis (e.g. meta-analysis) was conducted.
	13f	Describe any sensitivity analyses conducted to assess robustness of the synthesized results.	N/A – no quantitative analysis (e.g. meta-analysis) was conducted.
Reporting bias assessment	14	Describe any methods used to assess risk of bias due to missing results in a synthesis (arising from reporting biases).	Given the predominantly pre-clinical and early clinical stages of testing US-BBBD for drug delivery, a bias assessment

Section and Topic	Item #	Checklist item	Location where item is reported
			was not possible.
Certainty assessment	15	Describe any methods used to assess certainty (or confidence) in the body of evidence for an outcome.	Given the predominantly pre-clinical and early clinical stages of testing US-BBBD for drug delivery, no methods were used to assess the certainty of the body of evidence. However, all included studies were either open-label randomised or non-randomised experimental trials.
**RESULTS**			
Study selection	16a	Describe the results of the search and selection process, from the number of records identified in the search to the number of studies included in the review, ideally using a flow diagram.	The results from the search and selection process was summarised in the 'Included Studies' paragraph and the flowchart, 'Figure 2' in the results section.
	16b	Cite studies that might appear to meet the inclusion criteria, but which were excluded, and explain why they were excluded.	Described in the flowchart ('Figure 2') in the results section
Study characteristics	17	Cite each included study and present its characteristics.	All included studies are cited in 'Table 1''Table 2' and 'Table S2 (Supplementary Materials).
Risk of bias in studies	18	Present assessments of risk of bias for each included study.	Given the predominantly pre-clinical and early clinical stages of testing US-BBBD for drug delivery, a bias assessment for each study was not possible.
Results of individual studies	19	For all outcomes, present, for each study: (a) summary statistics for each group (where appropriate) and (b) an effect estimate and its precision (e.g. confidence/credible interval), ideally using structured tables or plots.	N/A – no quantitative analysis (e.g. meta-analysis) was conducted. Results of extracted data is listed in 'Table S2' (Supplementary Materials), and summaries in 'Table 1.', 'Table 2.' and 'Table 4.'
Results of syntheses	20a	For each synthesis, briefly summarise the characteristics and risk of bias among contributing studies.	Given the predominantly pre-clinical and early clinical stages of testing US-BBBD for drug delivery, a bias assessment was not possible.
	20b	Present results of all statistical syntheses conducted. If meta-analysis was done, present for each the summary estimate and its precision (e.g. confidence/credible interval) and measures of statistical heterogeneity. If comparing groups, describe the direction of the effect.	N/A – no quantitative analysis (e.g. meta-analysis) was conducted.
	20c	Present results of all investigations of possible causes of heterogeneity among study results.	Given the predominantly pre-clinical and early clinical stages of testing US-BBBD for drug delivery, significant heterogeneity may exist between study results.
	20d	Present results of all sensitivity analyses conducted to assess the robustness of the synthesized results.	N/A – no quantitative analysis (e.g. meta-analysis) was conducted.
Reporting biases	21	Present assessments of risk of bias due to missing results (arising from reporting biases) for each synthesis assessed.	Given the predominantly pre-clinical and early clinical stages of testing US-BBBD for drug delivery, a bias assessment was not possible.
Certainty of evidence	22	Present assessments of certainty (or confidence) in the body of evidence for each outcome assessed.	Given the predominantly pre-clinical and early clinical stages of testing US-BBBD for drug delivery, no methods were used to assess the certainty of the body of evidence. However, all included studies were either open-label randomised or non-randomised experimental trials.
**DISCUSSION**			
Discussion	23a	Provide a general interpretation of the results in the context of other evidence.	This is discussed in the section 'Discussions and

Section and Topic	Item #	Checklist item	Location where item is reported
			Conclusions'.
	23b	Discuss any limitations of the evidence included in the review.	This is discussed in the section 'Discussions and Conclusions'.
	23c	Discuss any limitations of the review processes used.	This is discussed in the section 'Discussions and Conclusions'.
	23d	Discuss implications of the results for practice, policy, and future research.	This is discussed in the section 'Discussions and Conclusions'.
**OTHER INFORMATION**			
Registration and protocol	24a	Provide registration information for the review, including register name and registration number, or state that the review was not registered.	Given the nature of the available data, a systematic review registration number is not available, and the authors decided not to register it.
	24b	Indicate where the review protocol can be accessed, or state that a protocol was not prepared.	N/A – a review protocol was not prepared
	24c	Describe and explain any amendments to information provided at registration or in the protocol.	Given the nature of the available data, a systematic review registration number is not available, and the authors decided not to register it.
Support	25	Describe sources of financial or non-financial support for the review, and the role of the funders or sponsors in the review.	Described in 'Funding section of this review.
Competing interests	26	Declare any competing interests of review authors.	Described in 'Funding section of this review.
Availability of data, code and other materials	27	Report which of the following are publicly available and where they can be found: template data collection forms; data extracted from included studies; data used for all analyses; analytic code; any other materials used in the review.	N/A – no template data collection forms were used and no quantitative analysis conducted.

From: Page MJ, McKenzie JE, Bossuyt PM, Boutron I, Hoffmann TC, Mulrow CD, et al. The PRISMA 2020 statement: an updated guideline for reporting systematic reviews. BMJ 2021;372:n71. doi: 10.1136/bmj.n71

For more information, visit: http://www.prisma-statement.org/
