# Supplementary material for: Ultrasound-Mediated Blood–Brain Barrier Disruption for Drug Delivery: A Systematic Review of Protocols, Efficacy, and Safety Outcomes from Preclinical and Clinical Studies
Source: Pharmaceutics. 2022 Apr 11;14(4):833. doi: 10.3390/pharmaceutics14040833 (PMC9029131; doi:10.3390/pharmaceutics14040833)
Supplement: Supplementary file 1 [file pharmaceutics-14-00833-s001.zip › pharmaceutics-1637448-supplementary/Revised Supplementary Materials/Table S2.pdf]

Table S2: Database of extracted protocol, ultrasound parameter, efficacy, and safety assessment data from all included articles.

MB: *microbubbles* f_c***:*** *transducer frequency;* ***PNP:*** *peak negative pressure;* ***MI:*** *mechanical index;* ***PD:*** *pulse duration;* ***PRF:*** *peak repetition frequency;* ***DC:*** *duty cycle;* ***SD:*** *sonication duration;* ***ISI:*** *Intersonication interval (period between sonications);* ***DS:*** *damage scores for assessing degree of haemorrhagic damage (0: no erythrocyte extravasation, 1: sparse erythrocyte extravasation; 2: petechial haemorrhage 3: extensive, haemorrhagic foci)*[1]*;* ***OLs:*** *overlapping locations;* ***NOLs*** *non-overlapping locations*

Numbers in bold indicate parameters varied and studied
"T =" indicates the time from sonication of safety data acquisition

First Author and Year Published	Subject	Transducer Type and Manufacturer	Transducer Application		MBs Administered		Ultrasound Parameters												Comparative Degree of BBB Disruption Observed	Reported Adverse Safety Outcomes			
			Target(s)	Intact Skull vs Craniotomy Application	Type	Dose	f_c (MHz)	PNP (MPa) *In situ*	MI	PD (ms)	PRF (Hz)	DC (%)	SD (s)	# Sonications	ISI (s)	# of US Sessions	Intersession Interval		Macroscopic	Histological	Biochemical	Behavioural and Electrophysiological
**Baghirov et al.** 2018 [2]	Mouse *(Metastatic Melanoma Model)*	Dual, single-element, focused transducers FUS Instruments™	Metastatic melanoma lesions	Intact	In-house, nanoparticle loaded MBs	5000 µL/kg	1.1	0.31-0.34	0.30-0.32	10	0.33	0.33	300	1	N/A	1	-	N/A	Some evidence of erythrocyte extravasation *T=immediately following sonication*	DS=1-2 *T=2 hours*	-	-
**Baseri et al.** 2010 [3]	Mouse	Single-element, focused transducer Riverside Research Institute™	Hippocampus	Intact	Definity® MBs	50 µL/kg	1.53	**0.30-0.98**	**0.24-0.79**	20	10	20	30	2	30	1	-	++	**0.46 + 0.61 + 0.75 MPa:** No evidence of gross haemorrhage **0.98 MPa:** Gross haemorrhagic and tissue damage *T=30 minutes*	**0.30 MPa:** DS=0-1 (few micro-vacuolations) **0.46 MPa:** DS=1-2 (more dark neurons + micro-vacuolations) **0.61 MPa:** DS=2 (more dark neurons + micro-vacuolations) **0.75 MPa:** DS=2 (significantly higher number of dark neurons + micro-vacuolations) No significant differences in adverse events @ 5 hours across all PNPs *T= 40 minutes and 5 hours*		
**Bing et al.** 2009 [4]	Mouse	Siemens Sonoline™ Antares VF10-5, unfocused, diagnostic scanner Siemens Medical Solutions™	Subcortical, cerebral locations	Intact	Definity® MBs	1200 µL/kg	5.7	**0.72 or 1.67**	**0.3 or 0.7**	20	10	20	30	3 *OLs*	30	1	-	+	-	**0.72 + 1.67MPa**: DS=0-1	-	-
						1200 µL/kg	5.7	3.10	1.3	0.00035	36	0.00126	30	5 *OLs*	30	1	-	N/A	-	DS=2	-	-
**Chen et al.** 2013 [5]	Mouse	Single-element, focused transducer Imasonic™	Hippocampus	Intact	**In-house, MBs**	4.8 x 10^7 MBs/mouse	1.5	**0.45** and **0.60**	**0.55** and **0.73**	6.7	5	3.35	3000	1	-	1	-	+	-	**0.60MPa**: DS=1; few dark neurons *T=1 hour*	-	-
					**In-house, acoustically active nanodroplets**	3 x 10^8 NDs/mouse	1.5	**0.45** and **0.60**	**0.55** and **0.73**	6.7	5	3.35	3000	1	-	1	-	+	-	No significant adverse events reported	-	-

**Chen et al.**<br>2014<br>[6]	Mouse	Single-element, focused transducer<br>Imasonic™	Hippocampus	Intact	Definity® MBs	50 µL/kg	1.5	**0.51 or 0.84**	**0.42 or 0.69**	1.3	5	0.65	660	1	-	1	**-**	++	-	**0.51MPa:** DS=0 **(all dextrans)**<br>**0.84MPa:** DS=0 **(3 and 70kDa)**; DS=1 **(500 and 2000kDa)**<br>*T=9 minutes*		
**Choi et al.**<br>2010<br>[7]	Mouse	Single-element, focused transducer<br>Riverside Research Institute™	Lateral thalamus and hippocampus	Intact	**In-house, 1-2µm MBs**	2.13 x 10 µL/mouse	1.5	**0.46-0.61**	**0.38-0.50**	20	10	20	30	2<br>*NOLs*	30	1	**-**	+-+++	-	**0.46MPa:** DS= 0-1<br>*T=30 minutes*	-	-
					**In-house, 4-5µm MBs**	2.13 x 10 µL/mouse	1.5	**0.30-0.61**	**0.24-0.50**	20	10	20	30	2<br>*NOLs*	30	1	**-**	+	-	**0.30MPa:** DS=1<br>*T=30 minutes*	-	-
**Choi et al.**<br>2011<br>[8]	Mouse	Single-element, focused transducer<br>Riverside Research Institute™	Lateral thalamus and hippocampus	Intact	Definity® MBs<br>***30 vs 180s infusion***	**10-250 µL/kg**	1.53	0.46	0.37	20	10	f20	660	1	-	1	-	+	-	No significant adverse events reported<br>***SD=30s****: T=18.5 minutes*<br>***SD=660s:*** *T=10 minutes*	-	-
						50 µL/kg	1.53	0.46	0.37	**0.1-30**	10	**0.1-30**	660	1	-	1	-	++				
						50 µL/kg	1.53	0.46	0.37	20	**1-25**	**10-50**	660	1	-	1	-	**0**				
						50 µL/kg	1.53	0.46	0.37	20	10	20	**30 or 660**	1	-	1	-	**0**				
**Choi et al.**<br>2011<br>[9]	Mouse	Single-element, focused transducer<br>Imasonic™	Lateral thalamus and hippocampus	Intact	Definity® MBs	50 µL/kg	1.5	0.51	0.42	0.0023<br>***Phasic pulses*** *with BRF* ***0.1-10 Hz***	**6250-100,000**	**1.44-23**	660	1	-	1	**-**	+	-	No significant adverse events reported	-	-
**Choi et al.**<br>2008<br>[10]	Mouse *(Healthy or APP/PS1 Alzheimer's model)*	r	Hippocampus	Intact	SonoVue® MBs	25 µL/mouse	1.53	0.6	0.49	20	10	20	30	2<br>*NOLs*	30	1	-	N/A	No significant adverse events reported<br>*T=immediately after and 22 hours after*	No significant adverse events reported<br>*T=1 hour*	-	-
**Choi et al.**<br>2010<br>[11]	Mouse	Single-element, focused transducer<br>Riverside Research Institute™	Hippocampus	Intact	SonoVue® MBs	25 µL/mouse	1.53	0.57	0.46	20	10	20	30	2<br>*OLs*	30	1	-	N/A	-	DS=1<br>*T=30 minutes*	-	-

**Englander et al.**<br>2021<br>[12]	Mouse<br>*(DIPG model)*	Single-element, focused transducer<br>Imasonic™	Tumour site	Intact	SonoVue® MBs<br>Or<br>Definity® MBs	100 µL	1.5	0.7	0.57	10	5	5	120	4<br>*NOLs*	Immediately after	**1 or 2**	-	N/A	No significant adverse events reported<br>*T <2 hours*	No significant adverse events reported<br>*T <5 hours*	-	**1 US session:** No changes in grip strength<br>**2 US sessions**: Transient bradycardia following MB administration; minor improvement in grip strength likely attributable to repeat testing<br>No physiological assessments for 1 US session
**Jordao et al.**<br>2013<br>[13]	Mouse<br>*(Healthy and TgCRND8 Azheimer's model)*	Single-element focused transducer<br>FUS Instruments™	Cortical targets	Intact	Definity® MBs	**40 µL/kg** (health mice)<br>**Or**<br>**80 µL/kg** (TgCRND8 mice)	0.5	0.3	0.42	10	1	1	120	4<br>*NOLs*	-	1	-	0	**-**	**All mice:** Increased astrogliosis @ day 4 and 15; increased glial activation @ 4 hours, peaking @ 4 days, resolving @ 15 days; increased IgG/IgM accumulation at sonicated site<br>*T=4 hours, 4 and 15 days*	**All mice:** Increased proteomic expression of Iba1 and GFAP, with same time points as seen in histology<br>**TgCRND8:** Reduction in amyloid-beta plaque burden within 4 days<br>*T=4 hours, 4 and 15 days*	-
**Kinoshita et al.**<br>2006<br>[14]	Mouse	Single-element, focused transducer<br>In-house	Midline location	Intact	Optison® MBs	50 µL	0.69	**0.6** or **0.8**	**0.72** or **0.96**	10	1	1	40	1	-	1	-	+	**0.8MPa:** Small, scattered petechiae<br>*T=4 hours*	**0.6MPa:** DS=1; some apoptotic cells<br>**0.8MPa:** DS=2; some apoptotic cells<br>*T=4 hours*	-	-
**Kinoshita et al.**<br>2006<br>[15]	Mouse	Single-element, focused transducer<br>In-house	Hippocampus and basal ganglia	Intact	Optison® MBs	**10 µL**	0.69	**0.6** or **0.8**	**0.72** or **0.96**	10	1	1	40	1	-	1	-	+	No significant adverse events reported<br>*T=3 hours*	**0.6MPa:** DS=0-1<br>*T=3 hours*	-	-
						**50 µL**	0.69	**0.8-1.1**	**0.96-1.32**	10	1	1	40	1	-	1	-	++	**1.1MPa:** Petechial haemorrhages<br>*T=3 hours*	**0.8MPa:** DS=1-2<br>**0.9 + 1.1MPa:** DS=2-3<br>*T=3 hours*	-	-
**Lapin et al.**<br>2020<br>[16]	Mouse	Single-element, focused transducer<br>FUS Instruments™	Hippocampus and frontal cortex location		Definity® MBs	**27-196 µL/kg**<br>***Long Infusion***	1.43	**0.56**	0.47	10	1	1	30	8<br>*NOLs*	30	1	-	+-++	**196 µL/kg:** T2 hypointense (haemorrhagic) lesions detected<br>*T=40 minutes*	**-**	-	-
						**27-391 µL/kg**<br>***Long Infusion***	1.43	**0.48**	0.4	10	1	1	30	8<br>*NOLs*	30	1	-	+	No significant adverse events reported<br>*T=40 minutes*	**-**	-	-

						**196-703 µL/kg**<br><br>***Long Infusion***	1.43	**0.39**	0.33	10	1	1	30	8<br><br>*NOLs*	30	1	-	+	**632 µL/kg + 703 µL/kg:** T2 hypointense (haemorrhagic) lesions detected<br><br>*T=40 minutes*	-	-	-
						**87** or **179 µL/kg**<br><br>***One bolus***	1.43	0.39	0.33	10	1	1	30	8<br><br>*NOLs*	120	1	-	+	**179 µL/kg:** T2 hypointense (haemorrhagic) lesions detected<br><br>*T=1 hour*	-	-	-
**Liu et al.**<br><br>2014<br><br>[17]	Mouse<br><br>*(Healthy and U87 Glioma model)*	Single-element, focused transducer<br><br>Sonic Concepts™	Tumoral site	Intact	SonoVue® MBs	4 µL/mouse	0.5	**0.3 or 0.7**	**0.42 or 0.99**	10	1	1	60	1	-	1	-	+++	-	**0.3MPa:** DS=0<br>**0.7MPa:** DS=3<br><br>*T=4 hours*	-	-
**McDannold et al.**<br><br>2017<br><br>[18]	Mouse	Single-element, focused transducer<br><br>In-house	Putamen + Thalamus	Intact	Optison® MBs<br><br>**Air vs O_2 carrier gas for anaesthetic**	200 µL/kg	0.69	**0.34-0.54**	**0.41-0.65**	10	1	1	120	2<br>*NOLs*	0	1	-	+	-	**0.34-0.36MPa:** DS=0-1<br>**0.46-0.54MPa:** DS=1-2<br><br>Air > O_2<br><br>*T=1-2 hours*	-	-
**McMahon et al.**<br><br>2020<br><br>[19]	Mouse	Single-element, focused transducer<br><br>In-house	Striatum and frontal cortex location	Intact	Definity® MBs	40 µL/kg	1.78	**0.93** or **1.87**	**0.7** or **1.4**	**0.005**<br><br>***(Phasic pulses*** *over a 10ms burst)* ***)***	**1250**	**0.625**	250	3	350	1	-	++	**1.87 MPa: SWI hypointense (haemorrhagic) lesions** detected<br><br>*T=6 minutes*	**0.93MPa:** DS=1-2<br><br>*T=4 hours*	-	-
							1.78	**0.33-0.93**	**0.25-0.70**	**10**<br><br>***(Tonic pulse)***	**0.5**	**0.5**	250	3	350	1	-		**0.93 MPa: SWI hypointense (haemorrhagic) lesions** detected<br><br>*T=6 minutes*	**0.33 and 0.40MPa:** DS=1<br><br>*T=4 hours*		
**Morse et al.**<br><br>2022<br><br>[20]	Mouse	Single-element, focused transducer<br><br>Sonic Concepts	Hippocampus	Intact	SonoVue® MBs<br><br>*30s infusion*	5000 µL/kg	1	0.53	0.53	**0.005**<br><br>***(Phasic pulses*** *over a 10ms burst)*	**1250**	**0.625**	250	1	-	1	-	+		**0.53 MPa:** DS=0<br><br>*T= 0 or 2 hours*	-	-
							1	**0.35** or **0.53**	**0.35** or **0.53**	**10**	**0.5**	**0.5**	250	1	-	1	-		-	**0.35 MPa:** DS=1<br>**0.53 MPa**: DS=1-2	-	-

										(Tonic pulse)												
**Morse et al.**<br>2019<br>[21]	Mouse	Single-element, focused transducer<br>Sonic Concepts™	Hippocampus	Intact	SonoVue® MBs<br>*30s infusion*	100 µL	1	0.35	0.35	**0.005**<br>***(Phasic pulses*** *over a 10ms burst**)***	**1250**	**0.625**	250	1	-	1	-	+	-	**Tonic pulses:** Greater erythrocyte extravasation	-	-
							1	0.35	0.35	**10**<br>***(Tonic pulse)***	**0.5**	**0.5**	250	1	-	1	-					
**Olumolade et al.**<br>2016<br>[22]	Mouse	Single-element, focused transducer<br>Manufacturer not specified	Striatum	Intact	In-house MBs *(first 3 weeks)*	1000 µL/kg	1.5	0.45	0.37	0.33	10	0.33	60	1	-	**2-10**	**Biweekly**	+	-	No significant adverse events reported<br>*T=1 month*	-	No changes in open field and rotarod performance tests
					Definity® MBs *(remaining weeks)*	1000 µL/kg	1.5	0.47	0.37	0.33	10	0.33	60	1	-	**2-6**	**Monthly**	+	-	No significant adverse events reported<br>*T=1 month*	-	No changes in open field and rotarod performance tests
						1000 µL/kg	1.5	1.5	1.22	6.67	10	6.67	60	1	-	1	**-**	++	-	DS=2; some neuronal loss<br>*T=1 day*	-	Transient hypoactivity, and loss of balance/coordination<br>Rotational affinity to side ipsilateral of sonication
**Omata et al.**<br>2019<br>[23]	Mouse	Single-element, focused transducer<br>Nepa Gene Co.™	Subcortical, cerebral location	Intact	**Inhouse MBs (C_3F_8)**	3 x10^9 MBs/kg	3.0	0.5 W/cm^2		50	10	50	180	1	-	1	**-**	+ -++	**-**	No significant adverse events reported<br>*T=1 day*	-	**-**
					**Inhouse MBs (C_4F_{10})**																	
					**Inhouse MBs (SF_6)**																	
					**Sonazoid® MBs**																	
					**SonoVue® MBs**																	
**Raymond et al.**<br>2007<br>[24]	Mouse	Single-element, focused transducer<br>In-house	Subcortical, cerebral location	Craniotomy	Optison® MBs	300 µL/kg	1.03	0.2	0.20	10	1	1	45-60	1<br>or<br>2<br>*NOLs*	Not specified	1	-	N/A	Superficial, petechial haemorrhages observed near craniotomy site (also observed in controls)	DS=2<br>*T=1 hour*	-	**-**
**Raymond et al.**<br>2008<br>[25]	Mouse<br>*(APPswe:PSEN1dE9 and*	Single-element, focused transducer<br>In-house	Hippocampus	Intact	Optison® MBs	30-50 µL/mouse	0.69	**0.67** or **0.8**	**0.81** or **0.96**	10	1	1	40-45	1	-	1	**-**	+	**-**	**0.67 MPa:** Scattered petechiae in 1/7 treated regions<br>**0.8 MPa:** Scattered petechiae in 2 regions	-	**-**

	PDAPP transgenic Alzheimer disease models)				Definity® MBs	10 µL/mouse														*T=3-6 hours*		
**Samiotaki et al.**<br>2012<br>[26]	Mouse	Single-element, focused transducer<br>Imasonic™	Hippocampus	Intact	In-house MBs<br>***(1-2µm)***	10^7 MBs/kg	1.53	**0.45** or **0.6**	**0.36** or **0.49**	0.07	10	0.07	60	1	-	1	-	+	-	Histological findings not reported<br>*T=7 days*		-
					In-house MBs<br>***(4-5µm)***	10^7 MBs/kg	1.53	**0.3-0.6**	**0.24-0.49**	0.07	10	0.07	60	1	-	1	-	+-++	-	Some cell loss detected at dentate gyrus and CA1<br>*T=7 days*	-	-
					In-house MBs<br>***(6-8µm)***	10^7 MBs/kg	1.53	**0.3-0.6**	**0.24-0.49**	0.07	10	0.07	60	1	-	1	-	++	-	Some cell loss detected at dentate gyrus and CA1<br>*T=7 days*	-	-
**Shen et al.**<br>2016<br>[27]	Mouse	Single-element, focused transducer<br>Manufacturer not specified	Subcortical, cerebral location	Intact	In-house MBs	**100 µL/kg**	1.28	**0.53** or **0.64**	**0.47** or **0.57**	7.8	1	0.78	60	1	-	1	-	++	-	**0.64MPa**: DS=1-2<br>*T=4 hours*	-	-
						**500 µL/kg**	1.28	**0.53** or **0.64**	**0.47** or **0.57**	7.8	1	0.78	60	1	-	1	-	++	-	**0.53 MPa:** DS=1<br>**0.64 MPa:** DS=2<br>*T=4 hours*	-	-
**Sierra et al.**<br>2017<br>[28]	Mouse	Single-element, focused transducer<br>Imasonic™	Striatum	Intact	In-house fluorescently labelled MBs	4 x 10^9 MBs/mouse	1.5	**0.60** or **0.75**	**0.49-0.61**	6.56	5	3.28	300	1	-	1	-	++	**0.60 MPa:** T2 hyperintensity (oedema) detected acutely<br>**0.75 MPa:** Transient T2 hyperintensity (oedema) reversing within 3-4 days<br>*T=20 minutes, and daily for 7 days (075 MPa only)*	**0.60MPa:** DS=1-2 (resolved after 1 week)<br>**0.75MPa:** DS=1-2 (resolved after 1 week) microglial activation 1 week after sonication<br>*All PNPs: T=1 week*<br>*0.6 + 0.75MPa: T=2 hours*	-	-
**Vlachos et al.**<br>2011<br>[29]	Mouse	Single-element focused transducer<br>Riverside Research Institute™	Hippocampus	Intact	In-house MBs<br>***(1-2µm)***	1x10^7 MBs/mouse	1.53	**0.45-0.60**	**0.36-0.49**	0.067	10	0.067	60	1	-	1	-	+	No significant adverse events reported<br>*T=2 hours*	**0.45 + 0.60 MPa:** No significant adverse events reported<br>*T=7 days*	-	-
					In-house MBs<br>**(4-5µm)**	x10^7 MBs/mouse	1.53	**0.30-0.60**	**0.24-0.49**	0.067	10	0.067	60	1	-	1	-	++	No significant adverse events reported<br>**0.60 MPa:** Hypointense artefact not correlated w/ histology	**0.30 MPa:** No significant adverse events reported<br>**0.45 MPa + 0.60 MPa:** Cell loss in granular layer; sparse dark neurons; no microhaemorrhagic change	-	-

					In-house MBs<br><br>***(6-8 µm)***	x10^7 MBs/ mouse	1.53	**0.30-0.60**	**0.24-0.49**	0.067	10	0.067	60	1	-	1	**-**	+++	No significant adverse events reported<br><br>**0.45 + 0.60 MPa:** Hypointense artefacts not correlated w/ histology	**0.30 + 0.45 MPa:** No significant adverse events reported<br><br>**0.60 MPa:** Cell loss; multiple dark neurons; no microhaemorrhagic change	-	**-**
**Wu et al.**<br><br>2014<br><br>[30]	Mouse *(4T1-luc2 breast cancer model)*	Single-element, focused transducer<br><br>Sonic Concepts™	Tumour site	Intact	Not administered		0.5	0.97	1.37	Continuous application			600	1	-	1	**-**	N/A	**-**	Significant number of apoptotic cells<br><br>*T=5 days*	-	**-**
**Zhang, D. et al.**<br><br>2020<br><br>[31]	Mouse<br><br>*(Healthy and PDCL glioma models)*	SonoCloud® single-element, unfocused transducer<br><br>CarThera™	Somatosensory cortex tumour site	Intact	Lumason® MBs	7500 µL/kg	1.05	0.3	0.29	23.8	1	2.38	120	1	-	**1**	**-**	N/A	**-**	DS=0-2 (few instances of white matter vacuolation)<br><br>*T=21 days*	-	**-**
						7500 µL/kg	1.05	0.3	0.29	23.8	1	2.38	120	1	-	**8**	Over 3 weeks	N/A	**-**	DS=0-2 (white matter vacuolation + hemosiderin laden macrophages)<br><br>*T=21 days*	-	**-**
**Zhao, B. et al.**<br><br>2018<br><br>[32]	Mouse	Vivid E9 M5S-D® multi element phase array, unfocused diagnostic transducer<br><br>GE Health™	Striatum	Intact	In-house MBs	**0.5-3x 10^7 MBs/ mouse**	1.5	0.98	0.8	Continuous delivery			180	1	-	1	-	+++	**-**	**0.5-1 x10^7 MB:** DS=0-1<br><br>**2 x10^7 MB:** DS=2 (ischaemic neurons, slight vacuolisation)<br><br>**3 x10^7 MB:** DS=2-3 (extensive neuronal loss)<br><br>*T=6 hours*	-	**-**
						1 x 10^7 MBs/ mouse	1.5	**0.49-0.98**	**0.4-0.8**	Continuous delivery			180	1	-	1	-	++	**-**	**MI=0.4:** DS=0<br>**MI=0.6:** DS=1<br>**MI=0.8:** DS=1<br><br>*T=6 hours*	-	**-**
						1 x 10^7 MBs/ mouse	1.5	0.98	0.8	Continuous delivery			**60-240**	1	-	1	-	+++	**-**	**SD=60s:** SD=1<br>**SD=120s:** SD=1<br>**SD=180s:** SD=1-2<br>**SD=240s:** DS=2 (slight vacuolization)<br><br>*T=6 hours*	-	**-**
**Ali et al.**<br><br>2018<br><br>[33]	Rat	Single-element focused transducer<br><br>In-house	Pons	Intact	Definity® MBs	20 µL/kg	1.68	1.1	0.85	10	1	1	120	2<br><br>*(left and right pons)*	300-420	1	-	-	**-**	No significant adverse events reported<br><br>*T≤6 hours*	-	No differences in coordination or grip strength between groups
**Aryal et al.**<br><br>2017<br><br>[34]	Rat	Single-element, focused transducer<br><br>In-house	Striatum	Intact	Definity® MBs	10 µL/kg	0.69	**0.55** or **0.81**	**0.66** or **0.98**	10	1	1	60	1	300+	1	**-**	+	**0.81MPa:** T2* cortical hypointense (petechiae) lesions detected in all animals<br><br>*T=Immediately after*	**0.55MPa:** DS=1<br>**0.81MPa:** DS=2<br><br>*T=1, 24, 48 and 72 hours*	-	-

**Aryal et al.**<br>2015<br>[35]	Rat<br>*(9L gliosarcoma model)*	Single-element, focused transducer<br>In-house	Striatal tumour site	Intact	Definity® MBs	10 µL/kg	0.69	0.55-0.81	0.66-0.98	10	1	1	60	1	300+	1	-	N/A	T2* hypointense (petechial) lesions detected<br>*T= Immediately after*	DS=0-1<br>*T=4 hours*	-	-
**Aryal et al.**<br>2015<br>[36]	Rat<br>*(9L Gliosarcoma model)*	Single-element, focused transducer<br>In-house	Striatum	Intact	Definity® MBs	10 µL/kg	0.69	**0.55** (sessions 1-2) and **0.81** (session 3)	**0.66** and **0.98**	10	1	1	60	1	300+	3	Weekly	N/A	**US + Doxorubicin:** T2* cortical hypointense (petechial) lesions detected<br>*T= 53 and 67 days*	**US:** DS=0<br>**US + Doxorubicin:** DS=1-3 (necrosis at worst); macrophage infiltration<br>*Necrosis corresponded with cortical hypointense spots observed on MRI*<br>*T=70 days*	-	-
**Aslund et al.**<br>2017<br>[37]	Rat	Single-element, focused transducer<br>Imasonic™	Striatum and thalamus	Intact	**Acoustic Cluster Therapy (ACT) MBs/microdroplet immersion** or<br>**Sonazoid® MBs**	1000 µL/kg	1<br>*(A)*	0.28	0.28	0.4	0.004	1000	30	2<br>***(A+B)***	0	1	-	++	No significant adverse events reported<br>*T=5 minutes*	**ACT MBs:** DS=1<br>*T=40 minutes*	-	-
							1<br>*(B)*	0.09	0.09	0.4	0.004	1000	600									
					Acoustic Cluster Therapy MB/microdroplet immersion	1000 µL/kg	1<br>*(A)*	0.28	0.28	0.4	0.004	1000	30	1	-	1	-		No significant adverse events reported	No significant adverse events reported	-	-
**Cho et al.**<br>2016<br>[38]	Rat	Single-element, focused transducer<br>FUS Instruments™	Striatum	Intact	Definity® MBs	20 µL/kg<br>*Infusion*	1	0.6-0.65	0.6-0.65	10	1	1	120	1	-	1	-	N/A	No significant adverse events reported<br>*T=Immediately after*	DS=0-1<br>*T=1 day*	-	-
**Chopra et al.**<br>2010<br>[39]	Rat	Single-element, focused transducer<br>In-house	Subcortical	Intact	Definity® MBs	10 µL/kg	1.08	**0.27-0.76**	**0.26-0.52**	10	1	1	300	4	300	1	-	++	**0.54 + 0.76MPa:** Irreversible T2 (haemorrhagic) lesions detected on MRI<br>*T=Immediately after*	**0.27 MPa:** DS=0-1<br>**0.39 MPa:** DS=0-3<br>**0.59 MPa:** DS=0-3<br>**0.78 MPa:** DS=2-3<br>*T=2-4 hours*		
							1.08	0.2 – 0.6 W *(power ramp)*		10	1	1	300	4<br>*NOLs*	300	3	Weekly	N/A	No significant adverse events reported	DS=0 (2/4 rats)<br>DS=1-3 (2/4 rats)	-	-

**Fan et al.**<br>2016<br>[40]	Rat	Single-element, focused transducer<br>Panametrics™	Tumour site	Intact	**In-house, SPIO-Dox loaded MBs**	2.5 x 10^{10} MBs/mL	1	**0.3 or 0.5**	**0.3 or 0.5**	5000 cycles	1	N/A	240	1	0	1	-	++	**-**	**0.3MPa**: DS=0<br>**0.5MPa**: DS=0	-	-
**Fan et al.**<br>2014<br>[41]	Rat	Single-element, focused transducer<br>Panametrics™	Striatum	Craniotomy	**In-house MBs**	1.5 x 10^7 MBs/kg	**1**	**0.3-1.5**	**0.3-1.5**	1	10	1	60	1	-	1	-	+-++	**1.5 MPa:** Oedematous + haemorrhagic areas detected on MRI<br>*T=2 hours*	*Histological findings not reported*<br>*T=6 hours*	-	-
					**SonoVue® MBs**	1.5 x 10^7 MBs/kg	**1**	**0.2-1.5**	**0.2-1.5**	1	10	1	60	1	-	1	-	+-+++	**1.5 MPa:** Oedematous + haemorrhagic areas detected on MRI	**0.2-0.5 MPa:** DS=1<br>**1 MPa:** DS= 1-2<br>**1.5 MPa:** DS=3; apoptotic cells detected	-	-
					**In-house MBs**	1.5 x 10^7 MBs/kg	**10**	**1.0-4.5**	**0.32-1.42**	1	10	1	60	1	-	1	-	+-++	No significant adverse events reported	**2-4.5 MPa:** DS=1	-	-
					**SonoVue® MBs**	1.5 x 10^7 MBs/kg	**10**	**2.0-4.5**	**0.63-1.42**	1	10	1	60	1	-	1	-	+	No significant adverse events reported	*Histological findings not reported*	-	-
					**In-house, carmustine loaded MBs**	9.89 x 10^9 MBs/rat	**10**	**2.5** and **4.5**	**0.79-1.42**	1	10	1	240	1	-	1	-	+-++	No significant adverse events reported	No significant adverse events reported	-	-
**Fan et al.**<br>2015<br>[42]	Rat	Single-element, focused transducer<br>Panametrics™	Striatum	Intact	In-house, carmustine loaded MBs	0.5 x 10^9 MBs/rat	**1**	**0.3-1.5**	**0.3-1.5**	1	10	1	240	1	-	1	-	+-+++	-	**1 MPa:** DS=1-2<br>**1.5 MPa:** DS=2<br>*T=5 minutes*	-	-
		10 MHz single-element, focused transducer<br>Sonic Concepts™					**10**	**1.49-4.49**	**0.47-1.42**	1	10	1	240	1	-	1	-	+-++	-	No significant adverse events reported	-	-
**Goutal et al.**<br>2018<br>[43]	Rat	Single-element, focused transducer<br>Imasonic™	Longitudinal area across cerebrum and cerebellum	Intact	SonoVue® MBs	200 µL/rat	1.5	0.6	0.49	3	1	0.3	300	1	-	1	-	N/A	No significant adverse events reported<br>*T=10 minutes*	**-**	-	-
**Han et al.**<br>2021<br>[44]	Rat	Single-element, focused transducer<br>FUS Instruments™	Striatum and thalamus	Intact	Definity® MBs<br>*Infusion*	20 µL/rat	1.1	**0.60-0.65** or **0.75-0.80**	**0.57-0.62** or **0.72-0.76**	10	1	1	120	1	-	1	-	+	**0.65 MPa:** Increased water movement on ADC<br>**0.80 MPa:** Increased and prolonged (up to 48 hours) water movement on ADC	**0.65 MPa:** DS 0<br>**0.80 MPa:** DS=2; prolonged and increased aquaporin-4 expression; neurodegeneration present<br>*T = 5 min, 24 and 48 hours*	-	-

																			T= 5 minutes, 24 and 48 hours			
**Huh et al.**<br>2020<br>[45]	Rat	Single-element, focused transducer<br>FUS Instruments™	**Striatum** and th**alamus**	Intact	Definity® MBs	10 µL/kg	1.13	0.65	0.61	10	1	1	120	2<br>*NOLs*	300	1	-	+	**-**	**Striatal targets:** DS=1<br>**Thalamic targets:** DS=2	-	-
**Jung et al.**<br>2019<br>[46]	Rat	Single-element, focused transducer<br>FUS Instruments™	Striatum and thalamus	Intact	Definity® MBs	20 µL/kg	1<br>*(B)*	0.6-0.72	0.6-0.72	10	1	1	120	2<br>*NOLs*	Simultaneously	1	-	+	No significant adverse events reported<br>*T=12 minutes*	No significant adverse events reported<br>*T=24 hours*	-	-
						No MBs	1<br>*(F)*	**0.5-2**	**0.5-2**	10	1	1	120	2<br>*NOLs*	Simultaneously	2<br>*(F + B)*	Not specified	+-++	**1 and 2MPa:** ΔT = ±1ºC compared to unsonicated regions	**2 MPa:** DS=2 (mild neuropil vacuolation)	-	-
						20 µL/kg	1<br>*(B)*	0.6-0.72	0.6-0.72	10	1	1	120									
						No MBs	1<br>*(F)*	1	1	10	1	1	120	2<br>*NOLs*	Simultaneously	2<br>***(F +B or B+F)***	Not specified	++	No significant adverse events reported	**B+F:** DS=3 (extensive neuropil vacuolation)	-	-
						20 µL/kg	1<br>*(B)*	0.6-0.72	0.6-0.72	10	1	1	120									
**Kobus et al.**<br>2016<br>[47]	Rat	Single-element, focused transducer<br>In-house	Striatum	Intact	Definity® MBs	10 µL/kg	0.69	**0.66-0.8**	**0.79-0.96**	10	1	1	60	3<br>*OLs*	120+	6	Weekly	++	Permanent abnormalities (e.g. hypointense/hyperintense spots, enlarged ventricles) detected, via T2 W MRI, in 12/15 rats<br>*T= Immediately after each session*	**DS=0-3** (necrosis)<br>Macrophage infiltration (14/15); hemosiderin deposits (11/15); iron/calcium mineralisation (5/15); cyst formation (4/15); astrogliosis/glial scarring (10/15)<br>*T=1-36 hours*	-	-
**Kovacs et al.**<br>2017<br>[48]	Rat	Single-element, focused transducer<br>FUS Instruments™	Frontal cortex	Intact	Optison® MBs<br>*Infusion*	100 µL/kg	0.59	0.3	0.39	10	1	1	120	1	-	1	-	N/A	Hypointense T2* voxels, indicating labelled macrophage extravasation<br>*T=6 days*	DS=0; some apoptotic cells; evidence of neuronal loss + astrogliosis<br>CD68+ macrophage activation and infiltration<br>*T=1,6, and 24 hours*	Increased transcription of chemokines, cytokines and tropic factors associated with sterile inflammation and NF-kB pathway.<br>Increased protein expression of proinflammatory cytokines and heat-shock proteins<br>*=0.5,1,6,12, and 24 hours*	-

**Kovacs et al.**<br>2018<br>[49]	Rat	Single-element, focused transducer<br>FUS Instruments™	Frontal cortex/striatum and hippocampus	Intact	Optison® MBs<br>*Infusion*<br>*1 dose given for both sonications*	100 µL/kg	0.55	0.3<br>*(first 2 sessions)*<br>0.5<br>*(subsequent sessions)*	0.40<br>0.67	10	0.5-0.6	0.5-0.6	120	2<br>*NOLS*	300+	**1 or 6**	Weekly	+	**1 US session:** few hypointense T2* lesions (microhaemorrhage)<br>**6 US sessions:** multiple hyperintense T2 lesions (astrogliosis) + enlarged ventricles; hypointense T2* lesions (micro-haemorrhages)<br>*T=7 and 13 weeks*	**1 US session**: DS=1; astrogliosis; iron deposition; slight CD68+ macrophage activation and infiltration; mild neurogenesis<br>**6 US sessions**: DS=1; astrogliosis; iron deposition; extensive CD68+ macrophage activation and infiltration; moderate neurogenesis<br>*T=7 and 13 weeks*	**6 US sessions:** hyperphosphorylated tau in frontal lobe sonications	-
**Liu et al.**<br>2009<br>[50]	Rat	Single-element, focused transducer<br>Imasonic™	Frontal lobe	Intact	None	-	1.5	**1.1-3.5**	**0.90-2.86**	10	1	1	30	1	-	1	-	++	**1.1MPa:** No T2* hypointensities (haemorrhagic lesions)<br>**1.9-3.5MPa:** Increasing area T2* hyperintensities (haemorrhagic lesions)<br>*T=21 and 48 minutes*	**1.1MPa:** DS=0-1<br>**1.9-3.5MPa:** Worsening haemorrhagic change<br>*T=1 hour*	-	-
**Liu et al.**<br>2010<br>[51]	Rat<br>*(Healthy and C6 Gliblastoma Models)*	Single-element, focused transducer<br>Imasonic™	Frontal lobe and striatum	Intact	SonoVue® MBs	2.5 µg/kg<br>*Healthy Rats*	0.4	**0.45-1.35**	**0.71–2.13**	10	1	1	30	1	-	1	-	+++	**≥0.98 MPa:** Large, hypointense T2* lesions (intracerebral haemorrhaging)<br>*T= <30 minutes*	No significant adverse events reported<br>*T=4 hours*	-	-
						2.5 µg/kg<br>*C6 Rats*	0.4	0.62	0.98	10	1	1	30	1	-	1	-	N/A	No significant adverse events reported	**DS=2** (areas of gliosis, infiltrating inflammatory cells)	-	-
**Liu et al.**<br>2010<br>[52]	Rat	Single-element, unfocused transducer<br>K-Sonic Inc. ™	Cerebrum<br>***Fixed*** *application*	Intact	SonoVue® MBs	25 µL/kg	0.028	0.8	4.78	10	1	1	**240 - 600**	1	-	1	-	+++	-	**SD=240s:** DS=0<br>**SD=360s:** DS=0-1<br>**SD=480s:** DS=1<br>**SD=600s:** DS=2 (mild necrosis)	-	-
						25 µL/kg	0.028	0.8	4.78	**10-100**	1	**1-10**	360	1	-	1	-	++	-	**50ms:** DS-1<br>**100ms:** DS=2<br>*T=4-6 hours*	-	-
			Cerebrum<br>***Scanning*** *application*	Intact		25 µL/kg	0.028	0.8	4.78	**50** or **100**	1	**5-10**	360	1	-	1	-	+++	-	**100ms:** DS=1	-	-
**Liu et al.**<br>2008	Rat	Single-element, focused transducer	Frontal lobe	Craniotomy	SonoVue® MBs	25 µL/kg	1.5	**0.55-4.9**	**0.45-4.0**	10	1	**1**	30	1	-	1	-	+++	**≥1.9 MPa:** SWI hypointensities (haemorrhagic)	**0.55 MPa:** DS=0<br>**0.78 MPa:** DS=0-1<br>**1.1 MPa:** DS=0-1<br>**1.9 MPa:** DS=0-2	-	-

[53]		Imasonic™																	**4.9MPa:** extensive haemorrhaging<br>*T=1 and 8 days*	**2.45 MPa:** DS=0-2; apoptotic cells detected<br>**3.47 MPa:** DS=0-2; apoptotic cells detected<br>**4.9 MPa:** DS=0-3 (extensive neuropil vacuolation, necrosis)**;** apoptotic cells detected<br>*T=4-6 hours*		
**Liu et al.**<br>2010<br>[54]	Rat	Single-element, focused transducer<br>Imasonic™	Frontal lobe	Craniotomy	SonoVue® MBs	25 µL/kg<br>*(1 dose per 3 sonications)*	1.5	**1.10**	0.90	10	1	1	30	**6**<br>*OLs*	<5<br>(300+ between 3rd and 4th)	1	-	++	No significant adverse events reported<br>*T=10 minutes and continuously until 3 hours; 4 and 24 hours*	DS= 0-1; no macrophage infiltration	-	-
						25 µL/kg	1.5	**2.45**	2.00	10	1	1	30	**1**	-	1	-		T2* hypointense (haemorrhagic) lesion detected; macrophage infiltration detected via SPIOs	DS= 2-3; macrophage infiltration observed	-	-
**Marty et al.**<br>2012<br>[55]	Rat	Single-element, focused transducer<br>Imasonic™	Thalamus	Intact	SonoVue® MBs	200 µL/rat	1.5	0.45	0.37	3	10	3	60	1	-	1	-	N/A	No significant adverse events reported	-	-	-
**McDannold et al.**<br>2019<br>[56]	Rat	ExAblate Neuro® low frequency, 1024 element phase array, focused transducer<br>InSightec™ Ltd.	Cerebral locations	Intact	Definity® MBs	10 µL/kg	0.23	0.12-0.19	0.25-0.40	5	1.1	0.55	**30** *(1st)*<br>**75** *(2nd)*	2<br>*OLs*	120+	3	3 weeks	N/A	No significant adverse events reported<br>*T=48 or 72 hours*	**Small scar (<1mm)** in one rat<br>*T=3 weeks*	-	-
**Mcdannold et al.**<br>2020<br>[57]	Rat	ExAblate Neuro® low frequency, 1024 element phase array, focused transducer<br>InSightec™ Ltd.	Cerebral locations	Intact	Definity® MBs<br>*(No MBs for first 4 sonications)*	10 µL/kg	0.23	0.068-0.165	0.14-0.34	5	1.1	0.55	**30** *(First 4)*<br>**55** *(Last 4)*	4<br>*OLs*<br>*(Applied twice - first 4 no MBs; last 4 MBs)*	120+	3	3 weeks	N/A	Hyperintense T2 (oedema) lesions detected in striatum/cortex in half of all sessions; lesion; did not resolve by day 7 in 3 rats<br>*T= 1 and 7 days*	DS=1; additional pale stained scars found in the striatum of 4 rats (3 of which had the unresolved oedema)<br>*T=2 days*		
**Mcdannold et al.**<br>2011<br>[58]	Rat	Single element, piston, unfocused transducer<br>In-house	Putamen + thalamus	Intact	Definity® MBs	10 µL/kg	0.53	**0.13-0.31**	**0.18-0.43**	10	1	1	60	4<br>*NOLs*	300+	1	-	++	No significant adverse events reported<br>*T=4 hours*	**0.24-0.31MPa:** DS=2 (mild parenchymal damage)<br>*T=4 hours*	-	-

**Mcmahon et al.**<br>2017<br>[59]	Rat	Single-element, focused transducer<br>FUS Instruments™	Striatum + thalamus	Intact	Definity® MBs<br>*Infusion*	**10** or **100** µL/kg	0.55	0.19-0.20<br>*Power ramp*	0.26-0.27	10	1	1	120	3<br>*NOLs*	900+	1	-	++	No significant adverse events reported<br>*T=4 hours*	No significant adverse events reported<br>*T=6 hours*	**10 µL/kg MB**: no changes<br>**100 µL/kg MB:** increased expression of genes associated with NF-kB pathway	-
						100 µL/kg	0.55	**0.20** or **0.29**	**0.27** or **0.39**	10	1	1	120	3<br>*NOLs*	900+	1	-	+	**0.20 + 0.29MPa:** Transient hyperintense T2* (oedema) lesions; transient hypointense (microhemorrhage) T2 lesion detected via T2 MRI in one animal<br>*T=4 hours and 4 days*	**0.20MPa: DS=1-2** (leukocyte infiltration + evidence of some neuronal degeneration)<br>**0.29MPa: DS=1-2** (leukocyte infiltration + evidence of some neuronal degeneration+ few microglial nodules + few necrotic foci)<br>*T=4 days*	**0.20 + 0.29MPa: increase expression of genes** associated with NF-kB pathway	-
**Mcmahon et al.**<br>2020<br>[60]	Rat	Single-element, focused transducer<br>FUS Instruments™	Striatum + thalamus	Intact	**Definity® MBs**<br>**BG8774 (In-house) MBs**<br>**MSB4 (In-house MBs)**<br>*Infusion*	Gas volume equivalents to 20 µL/kg of Definity® given	0.58	**0.25-0.45**	**0.33 – 0.59**	10	0.5	0.5	260	1	-	1	-	+	-	**All MBs:** DS=1 @ 1-day post sonication; DS=0 @ 7-days post sonication<br>*T=1 and 7 days*	**MSB4**: Significantly increased transcription of proinflammatory genes: *Il!b, Ccl2 and Tnf*	-
**Mcmahon et al.**<br>2020<br>[61]	Rat	Single-element, focused transducer<br>FUS Instruments™	Dorsal hippocampus	Intact	Definity® MBs	20 µL/kg<br>*Infusion*	0.58	0.36-0.39 (triggering pressure)<br>*Maintained @ 50% of triggering pressure*	0.47-0.51	10	1	1	120	3<br>*NOLs*	300+	1	-	+<br>**Dexamethasone** appeared to reduce BBBD and reversed BBBD quicker	No significant adverse events reported<br>*T=15 minutes and 2 hours*	**Saline:** Evidence of astrogliosis + increased vascular density<br>**Dexamethasone:** No significant adverse events<br>*T=10 days*	**Saline:** Increased GFAP, ICAM1, MCP1, VEGF expression<br>**Dexamethasone:** No significant adverse events<br>*T=2 days*	-
**O'Reilly et al.**<br>2017<br>[62]	Rat	Single-element, focused transducer<br>In-house	Subcortical targets	Intact	Definity® MBs	20 µL/kg<br>*Infusion*	0.552	0.28	0.34	10	1	1	120	1<br>***(Single vs multi-point sonications)***	300+	1	-	0<br>*No difference observed between single point or multi-point sonications*	No significant adverse events reported<br>*T=6 and 24 (T2 only) hours*	Small foci of tissue injury on H/E in 1/5 rats<br>*T=48 hours*	-	-

**O'Reilly et al.**<br><br>2011<br><br>[63]	Rat	Single-element, focused transducer arranged as a hemispherical array<br><br>Imasonic™	Midline, subcortical targets	Intact	Definity® MBs	20 µL/kg<br><br>***Bolus***	1.18	0.54	0.50	0.003<br><br>*Phasic pulses in a 10ms burst*	**3,333 – 166,667**	**1- 50**	120	8<br><br>*NOLs*	300	1	-	+	**6µs:** 17/40 sonications w/ possible oedema<br>**60µs:** 2/10 sonications w/ possible oedema<br><br>**300µs:** 4/10 sonications w/ possible oedema<br><br>**300µs: 3**/10 sonications w/ possible oedema	Correlative with MRI findings<br><br>*T=2 hour*	**-**	-
						20 µL/kg<br><br>***Infusion***	1.18	0.54	0.50	0.003<br><br>*Phasic pulses in a 10ms burst*	166,667	50	300	8<br><br>*NOLs*	300	1	-	+	**Bolus:** 28/32 sonications w/ possible oedema<br><br>**Infusion:** 5/32 sonications w/ possible oedema	*Histological findings not clearly reported*	**-**	-
						20 µL/kg<br><br>***Infusion***	1.18	0.54	0.50	0.003<br><br>*Phasic pulses in a 10ms burst at* ***varying BRFs (0.2-2 Hz)***	166,667<br><br>*Burst repetition frequency*	50	300	8<br><br>*NOLs*	300	1	-	+	No significant adverse events reported	*Histological findings not clearly reported*	**-**	-
**Park et al.**<br><br>2017<br><br>[64]	Rat<br><br>(Healthy and 9L gliosarcoma model)	Single-element, focused transducer<br><br>In-house	Cerebral locations or tumour Site	Intact	Definity® MBs<br><br>*Healthy Rats*	10 µL/kg	0.69	0.72	0.87	10	1	1	60	**6**<br><br>*OLs*	120+	1	-	++	No significant adverse events reported	No significant adverse events reported<br><br>*T=4 hours*	**-**	-
					Definity® MBs<br><br>*9L Rats*	10 µL/kg	0.69	0.72	0.87	10	1	1	60	**5**<br><br>*OLs*	120+	2<br><br>***(Bilateral sonnications)***	-					
**Park et al.**<br><br>2012<br><br>[65]	Rat	Single-element, focused transducer<br><br>In-house	Striatum and hippocampus	Intact	Definity® MBs	10 µL/kg	0.69	0.6-0.72		10	1	1	60	2<br><br>*NOLs*	120s +	**1**	-	+	Transient, hypointense T2 (microhemorrhage) lesion detected in one animal<br><br>*T=serially imaged for 7.5 hours*	DS=0-1<br><br>*T=4 hours*	**-**	-

						10 µL/kg	0.69	0.68 and 0.72		10	1	1	60	2<br>*NOLs*	120s +	**2**	**10** or **120 mins**	+	No significant adverse events reported	**10-min intersession duration**: DS=0-1<br>**120-min intersession duration:** DS=0-1	-	-
**Shin et al.**<br>2018<br>[66]	Rat	Single-element, focused transducer<br>Sonic Concepts™ Inc.	Cerebrum	Intact	**Definity® MBs**	**20 or 100 µL/kg**	0.52	0.3		10	1	1	60	1	-	1	-	++	-	**100 µL/kg:** DS=1<br>*T=4 hours*	-	-
					**SonoVue® MBs**	30 µL/kg	0.52	0.3	0.42	10	1	1	60	1	-	1	-	+	-	DS=1	-	-
					Definity® MBs	20 µL/kg	0.52	**0.2-1.5**	**0.28-2.08**	10	1	1	60	1	-	1	-	+-+++	-	**0.6 MPa:** DS=2<br>**1.5 MPa:** DS=3 (significant parenchymal damage)	-	-
						20 µL/kg	**1.6**	**0.3** or **0.6**	**0.24** or **0.47**	10	1	1	60	1	-	1	-	+	-	DS=1	-	-
						20 µL/kg	0.52	0.3	0.42	**1-100**	1	**0.1-10**	60	1	-	1	-	+-++	-	**100ms:** DS=3 (parenchymal damage)	-	-
						20 µL/kg	0.52	0.3	0.42	10	**1-5**	**1-5**	60	1	-	1	-	+-++	-	No significant adverse events reported	-	-
						20 µL/kg	0.52	0.3	0.42	10	1	1	**30-300**	1	-	1	-	+-++	-	**120s:** DS=1<br>**300s:** DS=2	-	-
**Song et al.**<br>2017<br>[67]	Rat	Therapeutic Imaging Probe System (TIPS), multi-element phase array, focused transducer<br>Philips™	Striatum	Intact (skin incision made over sagittal suture)	Size isolated, **2µm diameter** MBs	**4x10^8 - 4x10^9** MBs/kg	1	0.5	0.5	1	100	10	300	2<br>*NOLs*	Unknown	1	-	+	No significant adverse events reported<br>*T=immediately after*	-	-	-
					Size isolated, **6µm diameter** MBs	**4x10^7-4x10^8** MBs/kg	1	0.5	0.5	1	100	10	300	2<br>*NOLs*	Unknown	1	-	++	**2 x 10^8 MBs/kg:** micro-haemorrhages visible macroscopically<br>**4 x 10^8 MBs/kg:** large haemorrhagic areas visible macroscopically	-	-	-
**Treat et al.**<br>2007<br>[68]	Rat	Single-element, focused transducer<br>In-house	Striatum + thalamus/hippocampus (2 locations per hemisphere)	Intact	Optison® MBs	100 µL/kg	1.5 or 1.7	**0.5-1.6**	**0.38-1.31**	10	1	1	30	**1**	-	1	-	+	No significant adverse events reported<br>*T=4 hours*	**1.1MPa:** DS=1 (hyperchromatic neurons)<br>*T=4 hours*	-	-
						**100 - 500 µL/kg**	1.5 or 1.7	1.1	0.84 or 0.90	10	1	1	120	**5**<br>***OL***	240-300	1	-	+	**200 and 500µL/kg:** macroscopic lesion (tissue damage) observed	**100µL/kg:** DS=1-2<br>**200µL/kg:** DS=2-3<br>**500µL/kg:** DS=3	-	-
**Tsai et al.**<br>2018	Rat	Single-element, focused transducer<br>Imasonic™	Basal ganglia	Intact	SonoVue® MBs	**150 µL/kg**	0.4	**0.30-0.89**	**0.47-1.40**	10	1	1	120	1	-	**2**<br>or<br>**3**	48hrs	++	-	**0.51MPa:** DS=1-2 (mild parenchymal damage + macrophage infiltration); astrogliosis	**0.89 MPa:** elevation in plasma fibrinogen<br>*T=8 days*	**0.89 MPa: transient hypoactivity, ataxia and tremor** observed in **2/10** rats

[69]																				**0.89MPa:** DS=3 (tissue necrosis + macrophage infiltration); astrogliosis<br><br>*T= 5 days*		No other reported adverse behaviour events
						**400 µL/kg**	0.4	**0.21** or **0.51**	**0.33** or **0.80**	10	1	1	120	1	-	**1-3**	**0 or 48 hrs**	++	-	**0.21MPa:** DS=0; apoptotic cells; astrogliosis<br><br>**0.51MPa:** DS=3; apoptotic cells; astrogliosis<br><br>Number of apoptotic cells **increased with US sessions**<br><br>*T=2 hours*	-	-
**Wei et al.**<br><br>2013<br><br>[70]	Rat<br><br>*(Healthy and 9L glioma models)*	Single-element, focused transducer<br><br>Imasonic™	Striatum	Intact	SonoVue® MBs	100 µL/kg	0.5	0.6	0.85	10	1	1	60	**1**	-	1<br><br>or<br><br>2	1 day	++	-	-	-	-
**Wu et al.**<br><br>2017<br><br>[71]	Rat	Single-element, focused transducer<br><br>Imasonic™<br><br>*Diagnostic US used to observe MB pharmacodynamics*	Various targets	Intact	SonoVue® MBs	**4x10^{7}** MBs/kg	**0.4**	**0.39-0.87**	**0.62-1.38**	10	1	1	120	1	-	1	-	++	**SonoVue® +1.38MPa:** Hypointense SWI (microhaemorrhage) lesions detected	**0.54MPa:** DS=1<br>**1.38MPa:** DS=2<br><br>*T=4 hours*	-	-
					Definity® MBs														**Definity® +1.38MPa:** Hypointense SWI (microhaemorrhage) lesions detected	**0.54MPa:** DS=1<br>**1.38MPa:** DS=2	-	-
					USphere® MBs														**USphere® +1.38MPa:** Hypointense SWI (microhaemorrhage) lesions detected	**0.54MPa:** DS=1<br>**1.38MPa**: DS=**2**	-	-
**Yang et al.**<br><br>2013<br><br>[72]	Rat	Single-element, focused transducer<br><br>Sonic Concepts™	Cerebrum	Intact	SonoVue® MBs	**150** or **300 µL/kg**	1	158 W/cm^{2}		50	1	5	60	**1**	-	1	-	++	-	**150µL/kg:** DS=1<br>**300µL/kg:** DS=1-2<br><br>*T=4 or 24 hours*	-	-
						150 µL/kg	1	158 W/cm^{2}		50	1	5	60	**2**<br><br>*OL*	1200	1	-	+++	-	DS=2	-	-
**Yang et al.**<br><br>2014<br><br>[73]	Rat<br><br>(F98 Glioma model)	Single-element, focused transducer<br><br>Sonic Concepts™	Tumour site	Intact	SonoVue® MBs	150 µL/kg	1.04	0.9		50	1	5	60	1	-	1	-	++	-	DS=1<br><br>*T=7 or 31 hours*	-	-
**Yang et al.**<br><br>2012	Rat	Single-element, focused transducer	Cerebrum	Intact	SonoVue® MBs	**150-450 µL/kg**	1	2.86 W		50	1	5	60	1	-	1	-	++	-	**300µL/kg:** DS=1-2; apoptotic cells (0.25% of regions)	-	-

[74]		Panametrics™																		**450μL/kg:** DS=2; apoptotic cells (1.15% of regions)<br><br>*T=24 hours*		
**Yang et al.**<br><br>2011<br><br>[75]	Rat	Single-element, focused transducer<br><br>Panametrics™<br><br>Non-implanted	Cerebrum	Intact	SonoVue® MBs	150 μL/kg	1	0.9	0.9	50	1	5	60	**1**<br><br>or<br><br>**2**	**1200s** or **2400s**	1	-	++	-	**1 Sonication:** DS=2 (inflammatory cell aggregation)<br><br>**2 Sonications (1200s or 2400s ISI):** DS=2 (inflammatory cell aggregation + mild vacuolation)<br><br>*T=immediately after*	-	-
**Yang et al.**<br><br>2012<br><br>[76]	Rat<br><br>(F98 Glioma Model)	Single-element, focused transducer<br><br>Panametrics™	Tumour site	Intact	SonoVue® MBs	300 μL/kg	1	5.72W		50	1	5	60	1<br><br>***Sonications administered 6, 8 and 12 days after tumour implantation***	-	1	-	++	-	DS=1-2<br><br>*T=30 minutes*	-	-
**Zhang, Y. et al.**<br><br>2016<br><br>[77]	Rat	8-element array, focused transducer<br><br>Imasonic™	Temporal lobe	Intact	In-house MBs	300 μL/kg	1.5	0.69	0.56	20	1	2	120	1	-	1	-	+	No significant adverse events reported<br><br>*T=immediately, 1 and 7 days*	No significant adverse events reported	-	-
**Beccaria et al.**<br><br>2013<br><br>[78]	Rabbit	SonoCloud® single-element**,** unfocused transducer<br><br>CarThera™	Cerebrum	Craniotomy	SonoVue® MBs	30 μL/rabbit	1.05	**0.3** and **0.5**	**0.29** and **0.49**	35	1	3.5	120	1	-	1	-	++<br>*(0.5 MPa)*	-	**0.3 MPa: DS=2**<br>**0.5 MPa: DS=2**	-	-
						30 μL/rabbit	1.05	0.5	0.49	35	1	3.5	**120** or **60**	1	-	1	-	++	-	**60s SD: DS=2**<br>**120s SD: DS=2**	-	-
						30 μL/rabbit	1.05	**0.3-0.8**	**0.29-0.78**	25	1	2.5	120	1	-	1	-	+++	**0.6 MPa: oedema observed via FLAIR MRI**	**0.3 MPa: DS=1**<br>**0.5 MPa: DS=2**<br>**0.8 MPa: DS=3**	-	-
						30 μL/rabbit	1.05	**0.5** and **0.8**	**0.49** and **0.78**	15	1	1.5	120	1	-	1	-	*Not reported*	-	**0.5 MPa: DS=2**<br>**0.8 MPa: DS=2**	-	-
						30 μL/rabbit	1.05	0.8	0.78	10	1	1	120	1	-	1	-	++	-	**DS=2**	-	-
**Chopra et al.**<br><br>2010<br><br>[39]	Rabbit	Single-element, focused transducer<br><br>In-house	Subcortical regions	Intact	Definity® MBs	10 μL/kg	1.08	0.38	0.37	10	1	1	**30-1200**	4<br>*NOLs*		1	-	++	**SD 300s ≤: T2 MRI lesions observed**	**SD 30s: DS=1**<br>**SD 180 s: DS=2-3**<br>**SD 300s: DS=1-3**<br>**SD 600s: DS=2-3**<br>**SD 1200s: SD=2-3**	-	-

**Hynyen et al.**<br>2005<br>[1]	Rabbit	Single-element, focused transducer<br>In-house	Cortical locations	Craniotomy	Optison® MBs	50 µL/kg	0.69	**0.4-3.1**	**0.49-3.73**	10	1	1	**20**	**1-6**<br>***NOLs***	300-600	1	-	+<br>*(0.4 -1.4 MPa)*	-	**0.4-0.5 MPa: DS=1**<br>**0.5-1.4 MPa: DS=2** (mild neutrophil infiltration + light vacuolation); **few apoptotic and ischaemic cells**<br>**2.3 MPa: DS=2-3** (mild to moderate parenchymal damage); **many apoptotic and ischaemic cells**<br>**3.1 MPa: DS=3** (necrosis); **many apoptotic and ischaemic cells**	-	-
**Hynyen et al.**<br>2006<br>[79]	Rabbit	Single-element, focused transducer<br>In-house	Subcortical, cerebral locations	**Craniotomy** or **intact**	Optison® MBs	50 µL/kg	0.26	**0.3-0.9**	**0.58-1.77**	10	1	1	20	**1-4**<br>*NOLs*	300	1	-	+-++<br>*(0.3 and 0.4 MPa)*	-	**0.4 MPa: DS=1-2** (mild neutrophil infiltration + vacuolization); **few apoptotic cells.**<br>Histological findings were **acute (4-24hrs** post sonication**)** and disappeared days after sonication.<br>***No histological findings reported for PNPs >0.4MPa***		
**McDannold et al.**<br>2006<br>[80]	Rabbit	Single-element, focused transducer<br>In-house	Thalamus	Craniotomy	Optison® MBs	50 µL/kg	0.26	**0.29-0.57**	**0.57-1.12**	10	1	1	20	4<br>*NOLs*	300+	1	-	+-++	-	**0.40 MPa: DS=1**<br>**0.57 MPa: DS=2**	-	-
**McDannold et al.**<br>2007<br>[81]	Rabbit	Single-element, focused transducer<br>In-house	Thalamus	Craniotomy	**Optison® MBs**	50 µL/kg	0.69	0.5	0.60	10	1	1	20	4<br>*NOLs*	300+	1	-	+	-	**DS=1-2**	-	-
					**Definity® MBs**	10 µL/kg	0.69	0.5	0.60	10	1	1	20	4<br>*NOLs*	300+	1	-		-	**DS=0-1**	-	-
					Definity® MBs	10 µL/kg	0.69	**0.4-1.5**	**0.48-1.81**	10	1	1	20	4<br>*NOLs*	300+	1	-	+-+++	**1.1 and 1.5 MPa:** T2 hyperintense spots (mild oedema) detected near microhemorrhages	**0.4 MPa:** DS=0-1<br>**0.8 MPa:** DS=1-2<br>**1.1 MPa:** DS=1-2<br>**1.5 MPa:** DS=2	-	-
**McDannold et al.**<br>2008<br>[82]	Rabbit	Single-element, focused transducer<br>In-house	Thalamus	Craniotomy	Optison® MBs	50 µL/kg	2.04	**0.69-2.3**	**0.48-1.61**	10	1	1	20	4<br>*NOLs*	300+	1	-	+	-	DS=1	-	-
**McDannold et al.**<br>2008	Rabbit	Single-element, focused transducer	Thalamus	Craniotomy	Optison® MBs	**50-250 µL/kg**	0.69	0.5	0.60	10	1	1	20	4<br>*NOLs*	300+	1	-	+	-	**DS=1-2** (minor parenchymal damage)	-	-
						50 µL/kg	0.69	**0.8-1.5**	**0.96-1.81**	0.1	1	0.01	20	4<br>*NOLs*	300+	1	-	+				

[83]		In-house				50 µL/kg	0.69	**0.5-1.1**	**0.60-1.32**	1	1	0.1	20	4 *NOLs*	300+	1	-	+		*No clear difference in histological findings between locations sonicated with varying parameters*		
						50 µL/kg	0.69	0.5	0.60	**0.1-10**	1	**0.01-1**	20	4 *NOLs*	300+	1	-	+				
						50 µL/kg	0.69	0.5	0.60	10	**0.5-5**	**0.5-5**	20	4 *NOLs*	300+	1	-	0				
**Mei et al.** 2009 [84]	Rabbit	Single-element, focused transducer In-house	Various cortical and subcortical locations	Craniotomy	SonoVue® MBs	30 µL/kg	1.1	6W/0.07cm^2		Continuous delivery			**6-10s**	2-4 *NOLs*	300-600	1	-	++	-	**6s: DS=1** **8s: DS=2** (sight vacuolation, and mild ischaemic changes) **10s: DS=3** (extensive vacuolation, tissue necrosis, neutrophil infiltration)	-	-
**Wang et al.** 2009 [85]	Rabbit	Single-element, focused transducer In-house	Various cortical and subcortical locations	Craniotomy	SonoVue® MBs	30 µL/kg	1.1	6W/0.07cm^2		Continuous delivery			6	2-3 *NOLs*	300+	1	-	++	-	-	-	-
**O'Reilly et al.** 2017 [86]	Dog *(Aged dogs with significant comorbidities)*	Single-element, focused transducer In-house	Various cortical and subcortical locations	Intact	Definity® MBs	20 µL/kg	0.28	**0.81-1**	**1.53-1.89**	10	0.33	0.33	180	**6** *NOLs*	300+	1	-	+	**Thermal damage observed on overlying muscle** attributed to technical error	-	-	No changes in neurological testing and mental status
						20 µL/kg	0.28	**0.6-1.2**	**1.13-2.27**	10	1	1	120	**9-13** *NOLs*	300+	**1**	-	+-++	-	DS=1	-	No changes in neurological testing and mental status
						20 µL/kg	0.28	**0.6-1.2**	**1.13-2.27**	10	1	1	120	**7-12** *NOLs*	300+	**4**	**Weekly**	+-++	*T2 and T2*W MRI results not reported*	DS=1	-	Vomiting and bloody urine observed after second session in 1/5 dogs
**Liu et al.** 2011 [87]	Pig	Single-element, unfocused transducer K-Sonic™ Inc.	Frontal Cortex	**Craniotomy**	SonoVue® MBs	50 µL/kg	0.028	0.8	4.78	**30** or **100**	1	**3** or **10**	300	1	-	1	-	**+-++**	**30ms: T2 hyperintense (oedema) lesions** detected **100ms: T2 hyperintense (oedema) + T2* hypointense (micro-haemorrhagic) lesions** detected	**30ms: DS=1; mild iron deposition** (hemosiderin) **100ms: DS=3** (parenchymal damage); **extensive iron deposition** (hemosiderin); **apoptotic cells**	-	-
				**Intact**		50 µL/kg	0.028	0.8	4.78	100	1	1	300	1	-	1	-	+	-	-	-	-
**Pelekanos et al.** 2018 [88]	Sheep	Single-element, focused ultrasound transducer Sonic Concepts™ Inc.	Cortical and hippocampal locations	Intact	Definity® MBs	10 µL/kg	0.286	0.42	0.79	**1** or **10**	2	**0.2** or **2**	120	5 or 6 *NOLs*	600+	1	-	+	$\boldsymbol{\Delta T = \leq 1.76°C}$ compared to unsonicated skull	-	-	-
							0.286	**0.42-0.72**	**0.79-1.35**	1	2	0.2	120	5 or 11 *NOLs*	600+	1	-	+	*Average number of petechiae per slice macroscopically detected* **0.42 MPa:** 0.63 **0.63 MPa:** 5.87	**0.63 MPa≤: DS=1-2; few ischaemic cells detected**	-	-

																			**0.72 MPa:** 8.74<br><br>$\Delta T = \leq 1.76°C$ compared to unsonicated skull			
**Yoon et al.**<br><br>2019<br><br>[89]	Sheep	Single-element, focused ultrasound transducer<br><br>Ultran Group™	Subcortical locations	Intact	Definity® MBs	10 µL/kg	0.25	**0.48** or **0.58**	**0.96** or **1.16**	10	1	1	120	1	-	1	-	+	**0.58 MPa: SWI hypointense (haemorrhagic) lesions** detected	**0.58 MPa: DS=2**	-	-
**Arvantis et al.**<br><br>2012<br><br>[90]	NHP	ExAblate 4000® 1024-element phase array, focused transducer<br><br>InSightec™ Ltd.	Lateral geniculate nucleus, amygdala, cingulate gyrus	Intact	Definity® MBs	20 µL/kg<br><br>*Infusion*	0.22	0.13-0.28	0.28-0.60	10	0.55	0.55	50	3<br><br>*NOLs*	25	**10**	**2 mins**	+	**T2* hypointense (micro-haemorrhagic) lesions** detected<br><br>*T=minutes after*	**DS=0-2**<br><br>*T=2 hours*	-	-
							0.22	0.1-0.3	0.21-0.64	10	0.55	0.55	50	3<br><br>*NOLs*	25	**16-40**	**Over 5-7 weeks**	+	**T2* hypointense (micro-haemorrhagic) lesions** detected	-	-	-
**Downs et al.**<br><br>2015<br><br>[91, 92]	NHP	Single-element, focused transducer<br><br>Sonic Concepts™	Caudate, putamen, thalamus	Intact	In-house MBs	2.5 x 10^8 MBs/kg	0.5<br>***Non-anaesthetised***	0.3	0.42	10	2	2	120	1	-	**9** *NHP A* or **10** *NHP B*	Not specified	+-++	**NHP 1: Transient T2 hyperintense (oedema) lesions** detected after o**ne session**	-	-	No changes to motor function<br><br>No EMG changes at temporalis muscle
				Intact		2.5 x 10^8 MBs/kg	0.5<br>***Anaesthetised***	0.3	0.42	10	2	2	120	1	-	**5** *NHP A* or **13** *NHP B*	Over 10 months	+	**NHP 1:** Transient T2 hyperintense (oedema) spots detected after the last 3 sessions	-	-	**Both NHPs:** Transient decrease in reaction times
						2.5 x 10^8 MBs/kg	0.5	**0.2-0.4**	**0.28-0.57**	10	2	2	120	1	-	**27**	Over 12 months	+	**0.4 MPa:** Transient T2 hyperintense (oedema) spots detected after the last session	-	-	**Both NHPs**: Transient decrease in reaction times
						2.5 x 10^8 MBs/kg	0.5	**0.2-0.275**	**0.28-0.39**	10	2	2	120	1	-	**18**	Over 20 months	++	-	-	-	Transient increase in reaction times
						2.5 x 10^8 MBs/kg	0.5	0.4	0.57	10	2	2	120	1	-	4	Over 4 months	+-++	-	-	-	Transient increase in reaction times
**Goldwirt et al.**<br><br>2016<br><br>[93]	NHP	Single-element, unfocused transducer<br><br>In-house	Motor cortex	Craniotomy	SonoVue® MBs	100 µL/kg	1.05	0.9	0.88	23.2	1	2.32	134	1	-	1	-	-	-	-	-	-

**Horodyckid et al.**<br>2017<br>[94]	NHP	SonoCloud® single-element**,** unfocused transducer<br>CarThera™	Motor cortex	Craniotomy	SonoVue® MBs	100 µL/kg	1.05	**0.6-0.8**	**0.59-0.78**	23.2	1	2.32	131	1	-	7	15 days	++	FLAIR hyperintense spots detected in subarachnoid space<br>(further T2* imaging ruled out subarachnoid haemorrhage)	Mild hemosiderin deposition observed in cortical region of one NHP	-	Transient tachypnoea observed in 20% of sonications, normal clinical and behavioural testing scores.<br>No EEG or SSEP alterations attributable post-sonication
**Marquet et al.**<br>2014<br>[95]	NHP	Single-element, focused transducer<br>Sonic Concepts™	Striatum	Intact	In-house MBs	500 µL/NHP	0.5	**0.2-0.3**	**0.28-0.42**	10	2	2	120	1	-	10 sessions	2 weeks	N/A	-	-	-	-
						500 µL/NHP	0.5	**0.2-0.3**	**0.28-0.42**	10	2	2	120	1	-	7 sessions	2 weeks	N/A	-	-	-	Grossly normal cognitive and motor behaviour
**Marquet et al.**<br>2011<br>[96]	NHP	Single-element, focused transducer<br>Riverside Research Institute™	Visual cortex, caudate, hippocampus	Intact	**In-house MBs**<br>*(1st session in NHP 1)*	500 µL/NHP	0.5	**0.3** or **0.45**	**0.42** or **0.64**	10	2	2	120	2<br>*NOLs at visual cortex*	Immediately after	2 sessions	Not specified	+	-	-	-	Grossly normal cognitive and motor behaviour
					**Definity® MBs**<br>*(2nd session in NHP 1)*	500 µL/NHP	0.5	**0.6**	**0.85**	10	2	2	120	1<br>*Hippocampus*	-							
					**In-house MBs**<br>*(NHP 2)*	500 µL/NHP	0.5	**0.6**	**0.85**	10	2	2	120	1<br>*Caudate*	-	1 session	-	+	T2 hyperintense (oedematous) lesions	-	-	Reversible contralateral arm weakness, resolving after four days.<br>Grossly normal cognitive behaviour
**McDannold et al.**<br>2012<br>[97]	NHP	ExAblate 4000® 1024-element phase array, focused transducer<br>InSightec™ Ltd	Thalamus, putmen, cingulate cortex, visual cortex, hippocampus	Intact	Definity® MBs	10 µL/kg<br>***Bolus***	0.22	**0.19-0.66**	**0.41-1.41**	10	1	1	**70**	1	Not specified	1-13	1 or 2 weeks	++	**0.36 MPa <:** T2* hypointense (haemorrhagic) lesions detected in 50%+ of sonicated sites<br>*T = immediately after*	**0.7 MPa:** DS=2<br>*T = 2-48 hours and 2 weeks*		No adverse behavioural outcomes, including in motor testing, visual task performance or visual acuity<br>*T= days after*
						20 µL/kg<br>***Infusion***	0.22	**0.19-0.7**	**0.41-1.49**	10	1	1	**50**	9<br>*NOLs*	25	5	Months					
**Pouliopoulos et al.**<br>2019<br>[98]	NHP	Single-element focused transducer<br>Sonic Concepts™	Thalamus and dorsolateral prefrontal cortex	Intact	Definity® MBs	10 µL/kg	0.25	0.2	0.4	10	2	2	120	1	-	1	-	-	No MRI evidence of haemorrhagic or oedematous change<br>*T= 1 hour*	-	-	-
**Wu et al.**<br>2016<br>[99]	NHP	Single-element focused transducer<br>Sonic Concepts™	Striatum	Intact	In-house MBs	2.5 x 10^8 MBs/kg	0.5	0.2-0.6	0.28-0.85	10	2	2	120	**1** or **2**	1200	4-24	Weekly	++	No MRI evidence of haemorrhagic or oedematous change<br>*T= 1 hour*	-	-	-
**Abrahao et al.**	Human	ExAblate 4000® 1024-element phase array,	Primary motor cortex	Intact	Definity® MBs	4 µL/kg	0.22	**6-10W**		2.6	30.3	7.88	90	3	Not specified	1	-		Transient, asymptomatic FLAIR hyperintense	-	-	Moderate adverse events related to the procedure **(headache/scalp pain**

2019<br><br>[100]	(ALS patients)	focused transducer<br><br>InSightec™ Ltd.	(corresponding to arm or leg region)			*Patient 1*													(oedematous) lesion detected in patient 4			due to stereotactic frame placement, **musculoskeletal pain)** were reported<br><br>No EEG changes reported.rea
						4 µL/kg<br><br>*Patient 2*	0.22	**4 and 8W**		2.6	30.3	7.88	90	2	Not specified	1	-					
						4 µL/kg<br><br>*Patient 3*	0.22	**8-10W**		2.6	30.3	7.88	90	4	Not specified	1	-					
						4 µL/kg<br><br>*Patient 4*	0.22	**4-7W**		2.6	30.3	7.88	90	3	Not specified	1	-					
**Anastasiadis et al.**<br><br>2021<br><br>[101]	Human (Infiltrating glioma patients)	ExAblate Neuro 2® 1024-element phase array, focused transducer<br><br>InSightec™ Ltd	Tumour site	Intact	Definity® MBs	4-5 µL/kg<br><br>*Up to 20 µL/kg*	0.23	**3.38-21.55 W**				8	90	Not specified	Not specified	1	-		Transient, T2* hypointense lesions detected in 50% of sonication sites	DS=0		
**Chen et al.**<br><br>2021<br><br>[102]	Human (Glioblastoma multiforme patients)	NaviFUS® multi-element phase array, focused transfucer<br><br>NaviFUS™ Corp.	Peritumoral site<br><br>*3x3 grid*	Intact	SonoVue® MBs	100 µL/kg<br><br>(max dose 4.8 mL)	0.5	**0.48-0.68**	**0.48-0.68**	10	9	9	120	**1-3**	Not specified	1	-	+-++	No MRI evidence of haemorrhagic or oedematous change	Lack of lymphocytic (CD4+, CD8+ and FOXP3+) and macrophagic (CD68+) response	**-**	2 serious adverse events: hypernatremia in n=1<br><br>36, other adverse events in n=5
**Gasca-Salas et al.**<br><br>2021<br><br>[103]	Human (Parkinson's disease demented patients)	ExAblate Neuro 2® 1024-element phase array, focused transducer<br><br>InSightec™ Ltd	Parieto-occipito-temporal junction	Intact	Luminity® MBs	4 µL/kg	0.22	**5-60 W**						**2-8**	Not specified	2	2-3 weeks		SWAN hypointensities detected upon 1 week in n=2<br><br>No alterations in FDG or Flutemetamol PET uptake	-	-	No impairments in any neuropsychological domains tested<br><br>No alterations in physical impairment
**Idbaidh et al.**<br><br>2019<br><br>[104]	Human<br><br>(Glioblastoma multiform patients)	SonoCloud® single-element, unfocused transducer<br><br>CarThera™	Tumour and peritumoral parenchyma	Craniotomy	SonoVue® MBs	100 µL/kg	1.05	**0.78-1.15**	**0.76-1.12**	23.8	**0.5** or **1**	**1.19** or **2.38**	**150-270**	1	-	**1-10**	Monthly	+	Peritumoral oedema detected on MRI after 2 sessions (0.78 MPa; 1.03 MPa)	--	-	Transient facial palsy occurred in 1 patient (0.90 MPa); resolving within 2 hours.<br><br>One death outside of glioblastoma progression occurred during the trial
**Lipsman et al.**<br><br>2018	Human<br><br>(Alzheimer's	ExAblate 4000® 1024-element phase array,	Dorsolateral prefrontal cortex	Intact	Definity® MBs	4 µL/kg	0.22	**7.5W**<br><br>**3-3.5W**		2	30	6	50	1 (1st session)	Not specified	**2**	1 month		Transient T2* hypointense (potential microhemorrhage) spots detected	-	-	Transient increase in Neuropsychiatric Inventory Questionnaire

[105]	patients)	focused transducer<br><br>InSightec™ Ltd.	(white matter)											2 (2nd session)<br><br>*NOLs*								(NPI-Q) score 1 month after second US session.
						4 µL/kg	0.22	**5-6W**<br><br>**4-7W**		2	30	6	50	1 (1st)<br><br>2 (2nd)<br><br>*NOLs*	Not specified	**2**	1 month		-	-	-	No adverse physical or behavioural outcomes
						4 µL/kg	0.22	**3W**<br><br>**2.5-3.5 W**		2	30	6	50	1 (1st session)<br><br>2 (2nd session)<br><br>*NOLs*	Not specified	**2**	1 month		-	-	-	No adverse physical or behavioural outcomes
						4 µL/kg	0.22	**3.5W**<br><br>**3.5-5W**		2	30	6	50	1 (1st session)<br><br>2 (2nd session)<br><br>*NOLs*	Not specified	**2**	1 month		Transient T2* hypointense (potential microhemorrhage) spots detected	-	-	No adverse physical or behavioural outcomes
**Mainprize et al.**<br><br>2019<br><br>[106]	Human<br><br>(Malignant glioma patients)	ExAblate 4000® 1024-element phase array, focused transducer<br><br>InSightec™ Ltd.	Tumour and peritumoral parenchyma	Intact	Definity® MBs	4 µL/kg	0.22	**5-9 W**		2.6	30.3	7.88	50	2	Not specified	**1**	-	-	-	-	-	Minor pain due to stereotactic frame placement reported in 2 patients
						4 µL/kg	0.22	**8-10W**		2.6	30.3	7.88	50	5	Not specified	**1**	-					
						4 µL/kg	0.22	**6-7.5W**		2.6	30.3	7.88	50	2	Not specified	**1**	-					
						4 µL/kg	0.22	**4-15W**		2.6	30.3	7.88	50	5	Not specified	**1**	-					
**Park et al,**<br><br>2020<br><br>[107]	Human (Glioblastoma multiforme patients)	ExAblate 4000® 1024-element phase array, focused transducer<br><br>InSightec™ Ltd	Peritumoral site<br><br>*3x3 grid*	Intact	Definity® MBs	4 µL/kg<br><br>(Max dose 20 µL/kg)	0.22			2.6	30.4	7.90	80 (average)	3.66 (average) *3x3 grid*	174	**3** *(n=1)*<br><br>or<br><br>**6** *(n=5)*	4 weeks	-	No MRI evidence of haemorrhagic or oedematous change	-	-	No cognitive or neurological deficits related to BBBD<br><br>1 adverse event related to temozolomide therapy

References

1. Hynynen, K., N. McDannold, N.A. Sheikov, F.A. Jolesz, and N. Vykhodtseva, *Local and reversible blood-brain barrier disruption by noninvasive focused ultrasound at frequencies suitable for trans-skull sonications.* Neuroimage, 2005. **24**(1): p. 12-20.
2. Baghirov, H., S. Snipstad, E. Sulheim, S. Berg, R. Hansen, F. Thorsen, Y. Morch, C.L. Davies, and A.K.O. Aslund, *Ultrasound-mediated delivery and distribution of polymeric nanoparticles in the normal brain parenchyma of a metastatic brain tumour model.* PLoS One, 2018. **13**(1): p. e0191102.
3. Baseri, B., J.J. Choi, Y.S. Tung, and E.E. Konofagou, *Multi-modality safety assessment of blood-brain barrier opening using focused ultrasound and definity microbubbles: a short-term study.* Ultrasound Med Biol, 2010. **36**(9): p. 1445-59.
4. Bing, K.F., G.P. Howles, Y. Qi, M.L. Palmeri, and K.R. Nightingale, *Blood-Brain Barrier (BBB) Disruption Using a Diagnostic Ultrasound Scanner and Definity in Mice.* Ultrasound in Medicine and Biology, 2009. **35**(8): p. 1298-1308.
5. Chen, C.C., P.S. Sheeran, S.Y. Wu, O.O. Olumolade, P.A. Dayton, and E.E. Konofagou, *Targeted drug delivery with focused ultrasound-induced blood-brain barrier opening using acoustically-activated nanodroplets.* J Control Release, 2013. **172**(3): p. 795-804.
6. Chen, H. and E.E. Konofagou, *The size of blood-brain barrier opening induced by focused ultrasound is dictated by the acoustic pressure.* J Cereb Blood Flow Metab, 2014. **34**(7): p. 1197-204.
7. Choi, J.J., J.A. Feshitan, B. Baseri, S. Wang, Y.S. Tung, M.A. Borden, and E.E. Konofagou, *Microbubble-size dependence of focused ultrasound-induced blood-brain barrier opening in mice in vivo.* IEEE Trans Biomed Eng, 2010. **57**(1): p. 145-54.
8. Choi, J.J., K. Selert, Z. Gao, G. Samiotaki, B. Baseri, and E.E. Konofagou, *Noninvasive and localized blood-brain barrier disruption using focused ultrasound can be achieved at short pulse lengths and low pulse repetition frequencies.* J Cereb Blood Flow Metab, 2011. **31**(2): p. 725-737.
9. Choi, J.J., K. Selert, F. Vlachos, A. Wong, and E.E. Konofagou, *Noninvasive and localized neuronal delivery using short ultrasonic pulses and microbubbles.* Proc Natl Acad Sci U S A, 2011. **108**(40): p. 16539-16544.
10. Choi, J.J., S. Wang, T.R. Brown, S.A. Small, K.E. Duff, and E.E. Konofagou, *Noninvasive and transient blood-brain barrier opening in the hippocampus of Alzheimer's double transgenic mice using focused ultrasound.* Ultrason Imaging, 2008. **30**(3): p. 189-200.
11. Choi, J.J., S. Wang, Y.S. Tung, B. Morrison, 3rd, and E.E. Konofagou, *Molecules of various pharmacologically-relevant sizes can cross the ultrasound-induced blood-brain barrier opening in vivo.* Ultrasound Med Biol, 2010. **36**(1): p. 58-67.
12. Englander, Z.K., et al., *Focused ultrasound mediated blood-brain barrier opening is safe and feasible in a murine pontine glioma model.* Sci Rep, 2021. **11**(1): p. 6521.
13. Jordao, J.F., et al., *Amyloid-beta plaque reduction, endogenous antibody delivery and glial activation by brain-targeted, transcranial focused ultrasound.* Exp Neurol, 2013. **248**: p. 16-29.
14. Kinoshita, M., N. McDannold, F.A. Jolesz, and K. Hynynen, *Noninvasive localized delivery of Herceptin to the mouse brain by MRI-guided focused ultrasound-induced blood-brain barrier disruption.* Proc Natl Acad Sci U S A, 2006. **103**(31): p. 11719-11723.

15. Kinoshita, M., N. McDannold, F.A. Jolesz, and K. Hynynen, *Targeted delivery of antibodies through the blood-brain barrier by MRI-guided focused ultrasound.* Biochem Biophys Res Commun, 2006. **340**(4): p. 1085-1090.
16. Lapin, N.A., K. Gill, B.R. Shah, and R. Chopra, *Consistent opening of the blood brain barrier using focused ultrasound with constant intravenous infusion of microbubble agent.* Sci Rep, 2020. **10**(1): p. 16546.
17. Liu, H.L., C.Y. Huang, J.Y. Chen, H.Y. Wang, P.Y. Chen, and K.C. Wei, *Pharmacodynamic and therapeutic investigation of focused ultrasound-induced blood-brain barrier opening for enhanced temozolomide delivery in glioma treatment.* PLoS One, 2014. **9**(12): p. e114311.
18. McDannold, N., Y. Zhang, and N. Vykhodtseva, *The Effects of Oxygen on Ultrasound-Induced Blood-Brain Barrier Disruption in Mice.* Ultrasound Med Biol, 2017. **43**(2): p. 469-475.
19. McMahon, D., L. Deng, and K. Hynynen, *Comparing rapid short-pulse to tone burst sonication sequences for focused ultrasound and microbubble-mediated blood-brain barrier permeability enhancement.* J Control Release, 2020.
20. Morse, S.V., A. Mishra, T.G. Chan, T.M.d.R. R, and J.J. Choi, *Liposome delivery to the brain with rapid short-pulses of focused ultrasound and microbubbles.* J Control Release, 2022. **341**: p. 605-615.
21. Morse, S.V., A.N. Pouliopoulos, T.G. Chan, M.J. Copping, J. Lin, N.J. Long, and J.J. Choi, *Rapid Short-pulse Ultrasound Delivers Drugs Uniformly across the Murine Blood-Brain Barrier with Negligible Disruption.* Radiology, 2019. **291**(2): p. 459-466.
22. Olumolade, O.O., S. Wang, G. Samiotaki, and E.E. Konofagou, *Longitudinal Motor and Behavioral Assessment of Blood-Brain Barrier Opening with Transcranial Focused Ultrasound.* Ultrasound Med Biol, 2016. **42**(9): p. 2270-82.
23. Omata, D., et al., *Effects of encapsulated gas on stability of lipid-based microbubbles and ultrasound-triggered drug delivery.* J Control Release, 2019. **311-312**: p. 65-73.
24. Raymond, S.B., J. Skoch, K. Hynynen, and B.J. Bacskai, *Multiphoton imaging of ultrasound/Optison mediated cerebrovascular effects in vivo.* J Cereb Blood Flow Metab, 2007. **27**(2): p. 393-403.
25. Raymond, S.B., L.H. Treat, J.D. Dewey, N.J. McDannold, K. Hynynen, and B.J. Bacskai, *Ultrasound enhanced delivery of molecular imaging and therapeutic agents in Alzheimer's disease mouse models.* PLoS One, 2008. **3**(5): p. e2175.
26. Samiotaki, G., F. Vlachos, Y.S. Tung, and E.E. Konofagou, *A quantitative pressure and microbubble-size dependence study of focused ultrasound-induced blood-brain barrier opening reversibility in vivo using MRI.* Magn Reson Med, 2012. **67**(3): p. 769-77.
27. Shen, Y., J. Guo, G. Chen, C.T. Chin, X. Chen, J. Chen, F. Wang, S. Chen, and G. Dan, *Delivery of liposomes with different sizes to mice brain after sonication by focused ultrasound in the presence of microbubbles.* Ultrasound in Medicine and Biology, 2016. **42**(7): p. 1499-1511.
28. Sierra, C., C. Acosta, C. Chen, S.Y. Wu, M.E. Karakatsani, M. Bernal, and E.E. Konofagou, *Lipid microbubbles as a vehicle for targeted drug delivery using focused ultrasound-induced blood-brain barrier opening.* J Cereb Blood Flow Metab, 2017. **37**(4): p. 1236-1250.
29. Vlachos, F., Y.S. Tung, and E. Konofagou, *Permeability dependence study of the focused ultrasound-induced blood-brain barrier opening at distinct pressures and microbubble diameters using DCE-MRI.* Magn Reson Med, 2011. **66**(3): p. 821-30.

30. Wu, S.K., C.F. Chiang, Y.H. Hsu, T.H. Lin, H.C. Liou, W.M. Fu, and W.L. Lin, *Short-time focused ultrasound hyperthermia enhances liposomal doxorubicin delivery and antitumor efficacy for brain metastasis of breast cancer.* Int J Nanomedicine, 2014. **9**: p. 4485-94.
31. Zhang, D.Y., et al., *Ultrasound-mediated Delivery of Paclitaxel for Glioma: A Comparative Study of Distribution, Toxicity, and Efficacy of Albumin-bound Versus Cremophor Formulations.* Clin Cancer Res, 2020. **26**(2): p. 477-486.
32. Zhao, B., Y. Chen, J. Liu, L. Zhang, J. Wang, Y. Yang, Q. Lv, and M. Xie, *Blood-brain barrier disruption induced by diagnostic ultrasound combined with microbubbles in mice.* Oncotarget, 2018. **9**(4): p. 4897-4914.
33. Alli, S., et al., *Brainstem blood brain barrier disruption using focused ultrasound: A demonstration of feasibility and enhanced doxorubicin delivery.* J Control Release, 2018. **281**: p. 29-41.
34. Aryal, M., K. Fischer, C. Gentile, S. Gitto, Y.Z. Zhang, and N. McDannold, *Effects on P-Glycoprotein Expression after Blood-Brain Barrier Disruption Using Focused Ultrasound and Microbubbles.* PLoS One, 2017. **12**(1): p. e0166061.
35. Aryal, M., J. Park, N. Vykhodtseva, Y.Z. Zhang, and N. McDannold, *Enhancement in blood-tumor barrier permeability and delivery of liposomal doxorubicin using focused ultrasound and microbubbles: evaluation during tumor progression in a rat glioma model.* Phys Med Biol, 2015. **60**(6): p. 2511-2527.
36. Aryal, M., N. Vykhodtseva, Y.Z. Zhang, and N. McDannold, *Multiple sessions of liposomal doxorubicin delivery via focused ultrasound mediated blood-brain barrier disruption: a safety study.* J Control Release, 2015. **204**: p. 60-69.
37. Aslund, A.K., S. Snipstad, A. Healey, S. Kvale, S.H. Torp, P.C. Sontum, C.L. Davies, and A. van Wamel, *Efficient Enhancement of Blood-Brain Barrier Permeability Using Acoustic Cluster Therapy (ACT).* Theranostics, 2017. **7**(1): p. 23-30.
38. Cho, H.S., H. Lee, M. Han, J.R. Choi, T. Lee, S. Ahn, Y. Chang, and J. Park, *Localised down-regulation of p-glycoprotein by Focused Ultrasound and Microbubbles induced Blood-Brain Barrier Disruption in Rat Brain* Journal of Therapeutic Ultrasound, 2016. **5 (Supplement 1)**: p. 16-17.
39. Chopra, R., N. Vykhodtseva, and K. Hynynen, *Influence of exposure time and pressure amplitude on blood-brain-barrier opening using transcranial ultrasound exposures.* ACS Chem Neurosci, 2010. **1**(5): p. 391-398.
40. Fan, C.H., Y.H. Cheng, C.Y. Ting, Y.J. Ho, P.H. Hsu, H.L. Liu, and C.K. Yeh, *Ultrasound/Magnetic Targeting with SPIO-DOX-Microbubble Complex for Image-Guided Drug Delivery in Brain Tumors.* Theranostics, 2016. **6**(10): p. 1542-56.
41. Fan, C.H., H.L. Liu, C.Y. Ting, Y.H. Lee, C.Y. Huang, Y.J. Ma, K.C. Wei, T.C. Yen, and C.K. Yeh, *Submicron-bubble-enhanced focused ultrasound for blood-brain barrier disruption and improved CNS drug delivery.* PLoS One, 2014. **9**(5): p. e96327.
42. Fan, C.H., C.Y. Ting, Y.C. Chang, K.C. Wei, H.L. Liu, and C.K. Yeh, *Drug-loaded bubbles with matched focused ultrasound excitation for concurrent blood-brain barrier opening and brain-tumor drug delivery.* Acta Biomater, 2015. **15**: p. 89-101.
43. Goutal, S., M. Gerstenmayer, S. Auvity, F. Caille, S. Meriaux, I. Buvat, B. Larrat, and N. Tournier, *Physical blood-brain barrier disruption induced by focused ultrasound does not overcome the transporter-mediated efflux of erlotinib.* Journal of Controlled Release, 2018. **292**: p. 210-220.

44. Han, M., H. Seo, H. Choi, E.H. Lee, and J. Park, *Localized Modification of Water Molecule Transport After Focused Ultrasound-Induced Blood-Brain Barrier Disruption in Rat Brain.* Front Neurosci, 2021. **15**: p. 685977.
45. Huh, H., et al., *A local difference in blood-brain barrier permeability in the caudate putamen and thalamus of a rat brain induced by focused ultrasound.* Sci Rep, 2020. **10**(1): p. 19286.
46. Jung, B., H. Huh, E.H. Lee, M. Han, and J. Park, *An advanced focused ultrasound protocol improves the blood-brain barrier permeability and doxorubicin delivery into the rat brain.* J Control Release, 2019. **315**: p. 55-64.
47. Kobus, T., N. Vykhodtseva, M. Pilatou, Y. Zhang, and N. McDannold, *Safety Validation of Repeated Blood-Brain Barrier Disruption Using Focused Ultrasound.* Ultrasound Med Biol, 2016. **42**(2): p. 481-92.
48. Kovacs, Z.I., S. Kim, N. Jikaria, F. Qureshi, B. Milo, B.K. Lewis, M. Bresler, S.R. Burks, and J.A. Frank, *Disrupting the blood-brain barrier by focused ultrasound induces sterile inflammation.* Proc Natl Acad Sci U S A, 2017. **114**(1): p. E75-e84.
49. Kovacs, Z.I., T.W. Tu, M. Sundby, F. Qureshi, B.K. Lewis, N. Jikaria, S.R. Burks, and J.A. Frank, *MRI and histological evaluation of pulsed focused ultrasound and microbubbles treatment effects in the brain.* Theranostics, 2018. **8**(17): p. 4837-4855.
50. Liu, H.L., P.H. Hsu, P.C. Chu, Y.Y. Wai, J.C. Chen, C.R. Shen, T.C. Yen, and J.J. Wang, *Magnetic resonance imaging enhanced by superparamagnetic iron oxide particles: usefulness for distinguishing between focused ultrasound-induced blood-brain barrier disruption and brain hemorrhage.* J Magn Reson Imaging, 2009. **29**(1): p. 31-8.
51. Liu, H.L., et al., *Blood-brain barrier disruption with focused ultrasound enhances delivery of chemotherapeutic drugs for glioblastoma treatment.* Radiology, 2010. **255**(2): p. 415-425.
52. Liu, H.L., C.H. Pan, C.Y. Ting, and M.J. Hsiao, *Opening of the blood-brain barrier by low-frequency (28-kHz) ultrasound: a novel pinhole-assisted mechanical scanning device.* Ultrasound Med Biol, 2010. **36**(2): p. 325-35.
53. Liu, H.L., Y.Y. Wai, W.S. Chen, J.C. Chen, P.H. Hsu, X.Y. Wu, W.C. Huang, T.C. Yen, and J.J. Wang, *Hemorrhage detection during focused-ultrasound induced blood-brain-barrier opening by using susceptibility-weighted magnetic resonance imaging.* Ultrasound Med Biol, 2008. **34**(4): p. 598-606.
54. Liu, H.L., Y.Y. Wai, P.H. Hsu, L.A. Lyu, J.S. Wu, C.R. Shen, J.C. Chen, T.C. Yen, and J.J. Wang, *In vivo assessment of macrophage CNS infiltration during disruption of the blood-brain barrier with focused ultrasound: a magnetic resonance imaging study.* J Cereb Blood Flow Metab, 2010. **30**(1): p. 177-186.
55. Marty, B., et al., *Dynamic study of blood-brain barrier closure after its disruption using ultrasound: a quantitative analysis.* J Cereb Blood Flow Metab, 2012. **32**(10): p. 1948-1958.
56. McDannold, N., Y. Zhang, J.G. Supko, C. Power, T. Sun, C. Peng, N. Vykhodtseva, A.J. Golby, and D.A. Reardon, *Acoustic feedback enables safe and reliable carboplatin delivery across the blood-brain barrier with a clinical focused ultrasound system and improves survival in a rat glioma model.* Theranostics, 2019. **9**(21): p. 6284-6299.

57. McDannold, N., Y. Zhang, J.G. Supko, C. Power, T. Sun, N. Vykhodtseva, A.J. Golby, and D.A. Reardon, *Blood-brain barrier disruption and delivery of irinotecan in a rat model using a clinical transcranial MRI-guided focused ultrasound system.* Sci Rep, 2020. **10**(1): p. 8766.
58. McDannold, N., Y. Zhang, and N. Vykhodtseva, *Blood-brain barrier disruption and vascular damage induced by ultrasound bursts combined with microbubbles can be influenced by choice of anesthesia protocol.* Ultrasound Med Biol, 2011. **37**(8): p. 1259-1270.
59. McMahon, D. and K. Hynynen, *Acute Inflammatory Response Following Increased Blood-Brain Barrier Permeability Induced by Focused Ultrasound is Dependent on Microbubble Dose.* Theranostics, 2017. **7**(16): p. 3989-4000.
60. McMahon, D., A. Lassus, E. Gaud, V. Jeannot, and K. Hynynen, *Microbubble formulation influences inflammatory response to focused ultrasound exposure in the brain.* Sci Rep, 2020. **10**(1): p. 21534.
61. McMahon, D., W. Oakden, and K. Hynynen, *Investigating the effects of dexamethasone on blood-brain barrier permeability and inflammatory response following focused ultrasound and microbubble exposure.* Theranostics, 2020. **10**(4): p. 1604-1618.
62. O'Reilly, M.A., O. Hough, and K. Hynynen, *Blood-Brain Barrier Closure Time After Controlled Ultrasound-Induced Opening Is Independent of Opening Volume.* J Ultrasound Med, 2017. **36**(3): p. 475-483.
63. O'Reilly, M.A., A.C. Waspe, M. Ganguly, and K. Hynynen, *Focused-ultrasound disruption of the blood-brain barrier using closely-timed short pulses: influence of sonication parameters and injection rate.* Ultrasound Med Biol, 2011. **37**(4): p. 587-94.
64. Park, J., M. Aryal, N. Vykhodtseva, Y.Z. Zhang, and N. McDannold, *Evaluation of permeability, doxorubicin delivery, and drug retention in a rat brain tumor model after ultrasound-induced blood-tumor barrier disruption.* J Control Release, 2017. **250**: p. 77-85.
65. Park, J., Y. Zhang, N. Vykhodtseva, F.A. Jolesz, and N.J. McDannold, *The kinetics of blood brain barrier permeability and targeted doxorubicin delivery into brain induced by focused ultrasound.* J Control Release, 2012. **162**(1): p. 134-142.
66. Shin, J., C. Kong, J.S. Cho, J. Lee, C.S. Koh, M.S. Yoon, Y.C. Na, W.S. Chang, and J.W. Chang, *Focused ultrasound-mediated noninvasive blood-brain barrier modulation: preclinical examination of efficacy and safety in various sonication parameters.* Neurosurg Focus, 2018. **44**(2): p. E15.
67. Song, K.H., A.C. Fan, J.J. Hinkle, J. Newman, M.A. Borden, and B.K. Harvey, *Microbubble gas volume: A unifying dose parameter in blood-brain barrier opening by focused ultrasound.* Theranostics, 2017. **7**(1): p. 144-152.
68. Treat, L.H., N. McDannold, N. Vykhodtseva, Y. Zhang, K. Tam, and K. Hynynen, *Targeted delivery of doxorubicin to the rat brain at therapeutic levels using MRI-guided focused ultrasound.* Int J Cancer, 2007. **121**(4): p. 901-7.
69. Tsai, H.C., C.H. Tsai, W.S. Chen, C. Inserra, K.C. Wei, and H.L. Liu, *Safety evaluation of frequent application of microbubble-enhanced focused ultrasound blood-brain-barrier opening.* Sci Rep, 2018. **8**(1): p. 17720.
70. Wei, K.C., et al., *Focused ultrasound-induced blood-brain barrier opening to enhance temozolomide delivery for glioblastoma treatment: a preclinical study.* PLoS One, 2013. **8**(3): p. e58995.
71. Wu, S.K., P.C. Chu, W.Y. Chai, S.T. Kang, C.H. Tsai, C.H. Fan, C.K. Yeh, and H.L. Liu, *Characterization of Different Microbubbles in Assisting Focused Ultrasound-Induced Blood-Brain Barrier Opening.* Sci Rep, 2017. **7**: p. 46689.

72. Yang, F.Y., C.C. Chen, Y.H. Kao, C.L. Chen, C.E. Ko, S.C. Horng, and R.C. Chen, *Evaluation of Dose Distribution of Molecular Delivery After Blood-Brain Barrier Disruption by Focused Ultrasound with Treatment Planning.* Ultrasound in Medicine and Biology, 2013. **39**(4): p. 620-627.
73. Yang, F.Y., C.E. Ko, S.Y. Huang, I.F. Chung, and G.S. Chen, *Pharmacokinetic changes induced by focused ultrasound in glioma-bearing rats as measured by dynamic contrast-enhanced MRI.* PLoS ONE, 2014. **9 (3) (no pagination)**(e92910).
74. Yang, F.Y. and P.Y. Lee, *Efficiency of drug delivery enhanced by acoustic pressure during blood-brain barrier disruption induced by focused ultrasound.* Int J Nanomedicine, 2012. **7**: p. 2573-2582.
75. Yang, F.Y., Y.S. Lin, K.H. Kang, and T.K. Chao, *Reversible blood-brain barrier disruption by repeated transcranial focused ultrasound allows enhanced extravasation.* J Control Release, 2011. **150**(1): p. 111-116.
76. Yang, F.Y., H.E. Wang, G.L. Lin, H.H. Lin, and T.T. Wong, *Evaluation of the increase in permeability of the blood-brain barrier during tumor progression after pulsed focused ultrasound.* Int J Nanomedicine, 2012. **7**: p. 723-30.
77. Zhang, Y., et al., *Non-Invasive, Focal Disconnection of Brain Circuitry Using Magnetic Resonance-Guided Low-Intensity Focused Ultrasound to Deliver a Neurotoxin.* Ultrasound in Medicine and Biology, 2016. **42**(9): p. 2261-2269.
78. Beccaria, K., et al., *Opening of the blood-brain barrier with an unfocused ultrasound device in rabbits.* J Neurosurg, 2013. **119**(4): p. 887-98.
79. Hynynen, K., N. McDannold, N. Vykhodtseva, S. Raymond, R. Weissleder, F.A. Jolesz, and N. Sheikov, *Focal disruption of the blood-brain barrier due to 260-kHz ultrasound bursts: a method for molecular imaging and targeted drug delivery.* J Neurosurg, 2006. **105**(3): p. 445-454.
80. McDannold, N., N. Vykhodtseva, and K. Hynynen, *Targeted disruption of the blood-brain barrier with focused ultrasound: association with cavitation activity.* Phys Med Biol, 2006. **51**(4): p. 793-807.
81. McDannold, N., N. Vykhodtseva, and K. Hynynen, *Use of Ultrasound Pulses Combined with Definity for Targeted Blood-Brain Barrier Disruption: A Feasibility Study.* Ultrasound in Medicine and Biology, 2007. **33**(4): p. 584-590.
82. McDannold, N., N. Vykhodtseva, and K. Hynynen, *Blood-brain barrier disruption induced by focused ultrasound and circulating preformed microbubbles appears to be characterized by the mechanical index.* Ultrasound Med Biol, 2008. **34**(5): p. 834-840.
83. McDannold, N., N. Vykhodtseva, and K. Hynynen, *Effects of acoustic parameters and ultrasound contrast agent dose on focused-ultrasound induced blood-brain barrier disruption.* Ultrasound Med Biol, 2008. **34**(6): p. 930-937.
84. Mei, J., Y. Cheng, Y. Song, Y. Yang, F. Wang, Y. Liu, and Z. Wang, *Experimental study on targeted methotrexate delivery to the rabbit brain via magnetic resonance imaging-guided focused ultrasound.* Journal of Ultrasound in Medicine, 2009. **28**(7): p. 871-880.
85. Wang, F., Y. Cheng, J. Mei, Y. Song, Y.Q. Yang, Y. Liu, and Z. Wang, *Focused ultrasound microbubble destruction-mediated changes in blood-brain barrier permeability assessed by contrast-enhanced magnetic resonance imaging.* J Ultrasound Med, 2009. **28**(11): p. 1501-1509.

86. O'Reilly, M.A., R.M. Jones, E. Barrett, A. Schwab, E. Head, and K. Hynynen, *Investigation of the Safety of Focused Ultrasound-Induced Blood-Brain Barrier Opening in a Natural Canine Model of Aging.* Theranostics, 2017. **7**(14): p. 3573-3584.
87. Liu, H.L., et al., *In vivo MR quantification of superparamagnetic iron oxide nanoparticle leakage during low-frequency-ultrasound-induced blood-brain barrier opening in swine.* J Magn Reson Imaging, 2011. **34**(6): p. 1313-1324.
88. Pelekanos, M., G. Leinenga, M. Odabaee, M. Odabaee, S. Saifzadeh, R. Steck, and J. Gotz, *Establishing sheep as an experimental species to validate ultrasound-mediated blood-brain barrier opening for potential therapeutic interventions.* Theranostics, 2018. **8**(9): p. 2583-2602.
89. Yoon, K., W. Lee, E. Chen, J.E. Lee, P. Croce, A. Cammalleri, L. Foley, A.L. Tsao, and S.S. Yoo, *Localized Blood-Brain Barrier Opening in Ovine Model Using Image-Guided Transcranial Focused Ultrasound.* Ultrasound Med Biol, 2019. **45**(9): p. 2391-2404.
90. Arvanitis, C.D., M.S. Livingstone, N. Vykhodtseva, and N. McDannold, *Controlled ultrasound-induced blood-brain barrier disruption using passive acoustic emissions monitoring.* PLoS One, 2012. **7**(9): p. e45783.
91. Downs, M.E., A. Buch, M.E. Karakatsani, E.E. Konofagou, and V.P. Ferrera, *Blood-Brain Barrier Opening in Behaving Non-Human Primates via Focused Ultrasound with Systemically Administered Microbubbles.* Sci Rep, 2015. **5**: p. 15076.
92. Downs, M.E., A. Buch, C. Sierra, M.E. Karakatsani, T. Teichert, S. Chen, E.E. Konofagou, and V.P. Ferrera, *Long-Term Safety of Repeated Blood-Brain Barrier Opening via Focused Ultrasound with Microbubbles in Non-Human Primates Performing a Cognitive Task.* PLoS One, 2015. **10**(5): p. e0125911.
93. Goldwirt, L., M. Canney, C. Horodyckid, J. Poupon, S. Mourah, A. Vignot, J.Y. Chapelon, and A. Carpentier, *Enhanced brain distribution of carboplatin in a primate model after blood-brain barrier disruption using an implantable ultrasound device.* Cancer Chemother Pharmacol, 2016. **77**(1): p. 211-6.
94. Horodyckid, C., et al., *Safe long-term repeated disruption of the blood-brain barrier using an implantable ultrasound device: a multiparametric study in a primate model.* J Neurosurg, 2017. **126**(4): p. 1351-1361.
95. Marquet, F., T. Teichert, S.Y. Wu, Y.S. Tung, M. Downs, S. Wang, C. Chen, V. Ferrera, and E.E. Konofagou, *Real-time, transcranial monitoring of safe blood-brain barrier opening in non-human primates.* PLoS One, 2014. **9**(2): p. e84310.
96. Marquet, F., Y.S. Tung, T. Teichert, V.P. Ferrera, and E.E. Konofagou, *Noninvasive, transient and selective blood-brain barrier opening in non-human primates in vivo.* PLoS One, 2011. **6**(7): p. e22598.
97. McDannold, N., C.D. Arvanitis, N. Vykhodtseva, and M.S. Livingstone, *Temporary disruption of the blood-brain barrier by use of ultrasound and microbubbles: safety and efficacy evaluation in rhesus macaques.* Cancer Res, 2012. **72**(14): p. 3652-3663.
98. Pouliopoulos, A.N., S.Y. Wu, M.T. Burgess, M.E. Karakatsani, H.A.S. Kamimura, and E.E. Konofagou, *A Clinical System for Non-invasive Blood-Brain Barrier Opening Using a Neuronavigation-Guided Single-Element Focused Ultrasound Transducer.* Ultrasound Med Biol, 2020. **46**(1): p. 73-89.
99. Wu, S.Y., C.S. Sanchez, G. Samiotaki, A. Buch, V.P. Ferrera, and E.E. Konofagou, *Characterizing Focused-Ultrasound Mediated Drug Delivery to the Heterogeneous Primate Brain In Vivo with Acoustic Monitoring.* Sci Rep, 2016. **6**: p. 37094.

100. Abrahao, A., et al., *First-in-human trial of blood-brain barrier opening in amyotrophic lateral sclerosis using MR-guided focused ultrasound.* Nat Commun, 2019. **10**(1): p. 4373.
101. Anastasiadis, P., D. Gandhi, Y. Guo, A.K. Ahmed, S.M. Bentzen, C. Arvanitis, and G.F. Woodworth, *Localized blood-brain barrier opening in infiltrating gliomas with MRI-guided acoustic emissions-controlled focused ultrasound.* Proc Natl Acad Sci U S A, 2021. **118**(37).
102. Chen, K.T., et al., *Neuronavigation-guided focused ultrasound for transcranial blood-brain barrier opening and immunostimulation in brain tumors.* Sci Adv, 2021. **7**(6).
103. Gasca-Salas, C., et al., *Blood-brain barrier opening with focused ultrasound in Parkinson's disease dementia.* Nat Commun, 2021. **12**(1): p. 779.
104. Idbaih, A., et al., *Safety and Feasibility of Repeated and Transient Blood-Brain Barrier Disruption by Pulsed Ultrasound in Patients with Recurrent Glioblastoma.* Clin Cancer Res, 2019. **25**(13): p. 3793-3801.
105. Lipsman, N., et al., *Blood-brain barrier opening in Alzheimer's disease using MR-guided focused ultrasound.* Nat Commun, 2018. **9**(1): p. 2336.
106. Mainprize, T., et al., *Blood-Brain Barrier Opening in Primary Brain Tumors with Non-invasive MR-Guided Focused Ultrasound: A Clinical Safety and Feasibility Study.* Sci Rep, 2019. **9**(1): p. 321.
107. Park, S.H., M.J. Kim, H.H. Jung, W.S. Chang, H.S. Choi, I. Rachmilevitch, E. Zadicario, and J.W. Chang, *Safety and feasibility of multiple blood-brain barrier disruptions for the treatment of glioblastoma in patients undergoing standard adjuvant chemotherapy.* J Neurosurg, 2020: p. 1-9.
